# Buffer Region Embedding for Hybrid Machine-Learned/Molecular-Mechanical Simulations in Complex Environments

**DOI:** 10.1021/acs.jcim.6c00924

**Published:** 2026-08-04

**Authors:** Michael Caspary, Radek Crha, Edgar Galicia-Andrés, Peter Poliak, Chris Oostenbrink

**Affiliations:** † Institute for Molecular Modeling and Simulation, 27270 BOKU University, 1190 Vienna, Austria; ‡ Christian Doppler Laboratory for Molecular Informatics in the Biosciences, BOKU University, 1190 Vienna, Austria; § Institute of Physical Chemistry and Chemical Physics, Faculty of Chemical and Food Technology, Slovak University of Technology in Bratislava, Radlinského 9, Bratislava 81237, Slovakia

## Abstract

Multiscale approaches combining machine-learned interatomic potentials with classical molecular mechanics force fields are emerging as a scalable alternative to QM/MM approaches, in which a quantum mechanical calculation is embedded in a molecular mechanics environment. They enable quantum-level accuracy for localized chemistry at substantially reduced cost. However, reliable coupling across region boundaries and application in heterogeneous environments remains a central challenge. Here, we extend the buffer region embedding strategy (BuRNN) for hybrid machine-learned interaction potentials/molecular mechanics (MLIP/MM) simulations in complex environments. The buffer region elevates the interactions of the inner region with its immediate surroundings to the MLIP level, whereas the interactions within the buffer region and with the outer region are still described at the MM level. We validate the approach across four test systems of increasing complexity: methanol/water mixtures benchmarked against experimental total X-ray structure factors, a functionalized fullerene designed to describe a covalent boundary between the buffer and outer region, aqueous heme b with an axial cysteine ligand to probe coordinative bond dissociation, and the resting-state of the cytochrome P450 1A2 enzyme. Across these systems, BuRNN reproduces experimental observables or the underlying QM reference data and yields stable dynamics, including challenging metal–ligand interactions. We compare explicit and implicit link-atom treatments at the buffer–outer boundary and find that explicit capping provides more robust uncertainty estimates, which is critical for active-learning model generation. These results establish BuRNN as a practical approach to perform MLIP/MM simulations in biomolecular systems.

## Graphical abstract

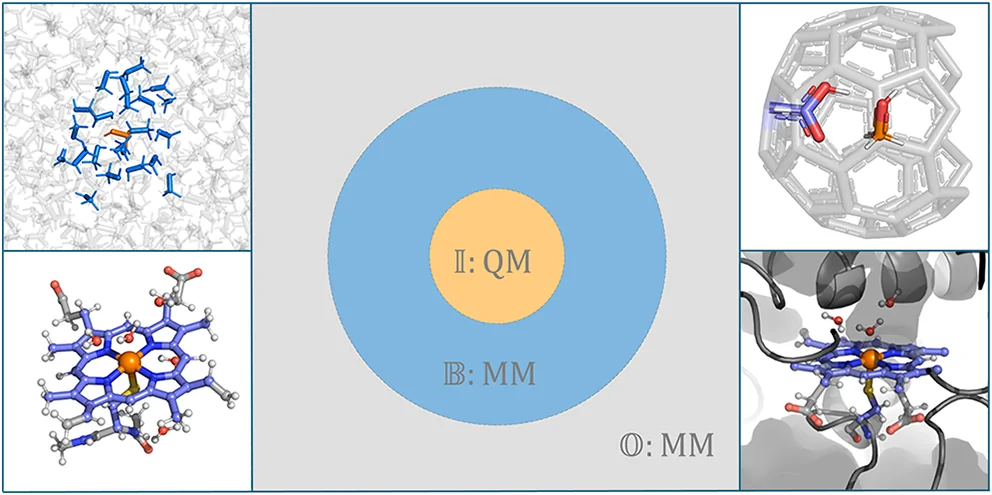

## 1 Introduction

Machine-learned interatomic potentials (MLIPs) have emerged as a highly transformative approach to predict quantum-mechanical (QM) quality forces from a small set of training configurations. The ability to learn the potential energy surface (PES) at the underlying reference level have been demonstrated repeatedly.^1^ MLIPs enable a significant speed-up in calculations and can therefore boost the accessible time scale of QM-quality simulations by orders of magnitude.

However, there are two underlying issues which should be considered if MLIPs of the early generation only learned interactions within a short-ranged local neighborhood.^1–3^ While this near-sightedness might be applicable for some specific scientific questions, a PES that lacks long-range interactions cannot accurately describe macromolecules, such as proteins in a condensed environment.^4,5^ Recent models incorporate long-range electrostatics either by directly learning specific partial charges, or by charge equilibration schemes.^6–8^ The former approach requires additional consideration when calculating electrostatic forces because the partial charges become a function of the coordinates.^9^ In the latter, charge equilibration approach, environment-dependent charges are obtained on-the-fly by minimizing a quadratic electronegativity functional under a total-charge constraint, such that the electrostatic energy and its forces are evaluated self-consistently without *ad-hoc* correction terms. These methods may partially alleviate the near-sightedness by facilitating the addition of classical long-range interactions. However, they come with an increased computational overhead. Full-MLIP simulations of large systems remain more expensive than classical molecular mechanics (MM).^10^

MLIP/MM approaches effectively circumvent the issue related to the nonlocality by retaining long-range effects through a coupling scheme, where regions of interactions are defined. An inner region ( I ) is described by the MLIP and an outer region ( O ) by a classical force field, which can be done with different approximations.^11^ This approach is less resource intensive, especially when one is interested in a specific part of the system, such as the active site of a protein. In such a setup I usually consists of the chemically active region, while the benefit of efficient and well-parametrized force fields for biomolecular systems can be fully leveraged for O . The advantage of a computationally cheap evaluation of the classical energy and forces can be used to calculate the rest of the system, where a coarser description of the energy landscape is typically justified.^12^ The developments in MLIPs have shown that MLIP/MM calculations are becoming a cost-effective and accurate alternative to well-established QM/MM methods.^9,13,14^

In QM/MM methods, a small part of the system (10^1^ to 10^2^ atoms) is typically treated by an accurate, but expensive QM method, while the rest of the system is treated by a classical force field. When partitioning a system into two regions, it is crucial to handle the coupling between these regions appropriately. This coupling can be treated at various levels of mutual interaction:^15^

- Mechanical Embedding is the simplest and least accurate procedure, Here the interactions between I and O are described with point charges from the underlying MM force field.
- Electrostatic Embedding incorporates the MM charge distribution in the QM Hamiltonian, such that the electronic structure of I is polarized by O . However, the MM charge distribution itself remains fixed and does not respond to the QM density.
- Polarizable embedding extends electrostatic embedding by introducing responsive MM degrees of freedom, such as fluctuating charges,^16^ induced dipoles,^17^ or combined fluctuating-charge/fluctuating-dipole models.^18^ These allow the MM environment to respond self-consistently to the QM density and, depending on the model, to other MM polarization sites. This provides a more complete description of mutual QM/MM and MM/MM polarization effects, but increases the computational cost because the coupled polarization response has to be solved self-consistently.

Herein, we aim to standardize and expand the QM/MM methodology Buffer Region Neural Network (BuRNN) to more complex systems. The BuRNN approach has successfully described the dynamics of a single solvated Fe­(III) in water and was applied to perform relative hydration free energy calculations between methanol and methane.^19,20^ In contrast to electrostatic embedding ML/MM approaches such as EMLE,^13,14^ which aim to reproduce the electrostatic polarization of the I region by the O through learned static and induced electrostatic interactions, or related strategies that learn QM/MM energies and forces within an electrostatic embedding framework,^21^ BuRNN focuses on the short-range QM/MM interface itself by introducing an explicit buffer region ( B ). This B serves to elevate the interaction energies of I with B to the QM level, whereas the interactions within B are described by MM, see Figure 1 [A]. The total potential energy *V*_tot_ consists of the QM energy of I and B as $V_{I+B}^{\mathrm{QM}}$ , the MM energy coupling of O to all other regions by $V_{O\left(I,B,O\right)}^{\mathrm{MM}}$ and additionally the difference between two B terms, evaluated at both the QM and MM level as $V_{B}^{\mathrm{MM}}-V_{B}^{\mathrm{QM}}$ , which helps to smooth the transition between the I and O . The total potential energy is hence defined in eq 1 as

1 $$V_{\mathrm{tot}}=V_{I+B}^{\mathrm{QM}}-V_{B}^{\mathrm{QM}}+V_{B}^{\mathrm{MM}}+V_{O\left(I,B,O\right)}^{\mathrm{MM}}$$

**Figure 1:**
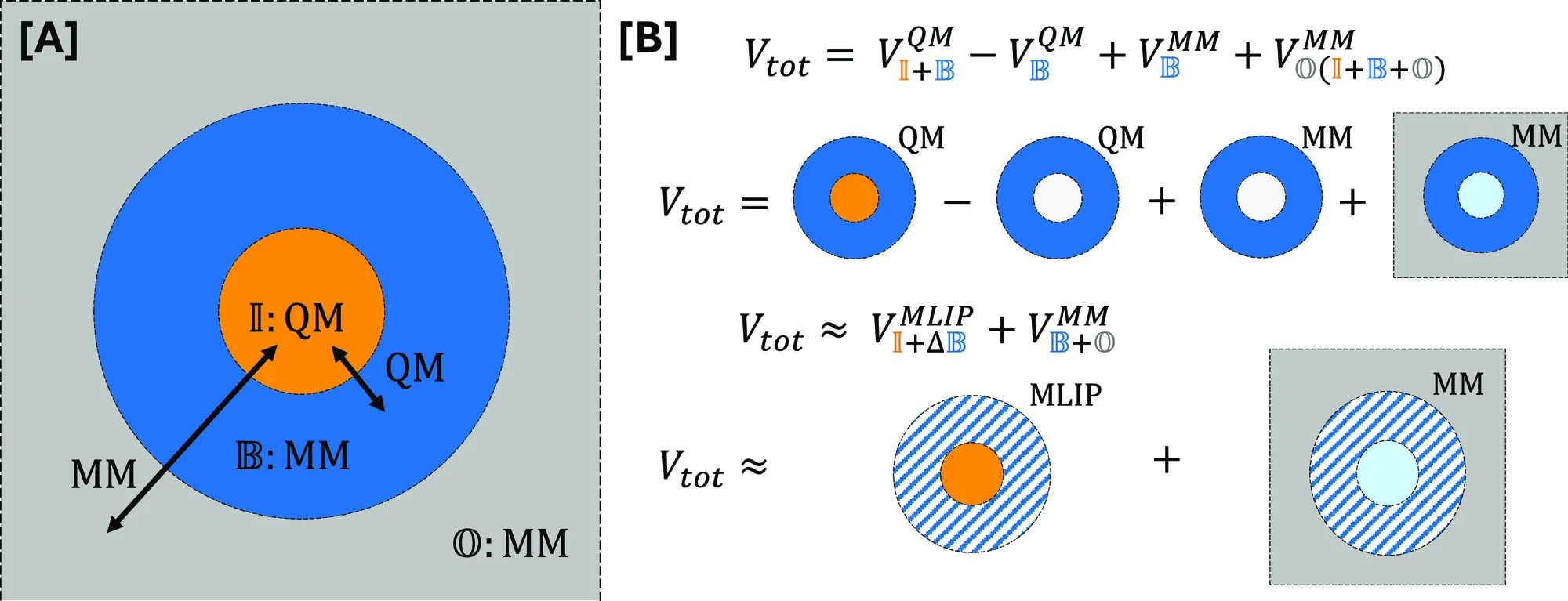
BuRNN approach. [A] shows a schematic approach, where I refers to the inner region, which is fully treated at the QM level. B refers to the buffer region, which is treated by QM and MM simultaneously. O refers to the outer region which is treated by MM. [B] shows the calculation of the total energies in the buffer region embedding, where the subscripts refer to the respective regions and the superscripts to the method of calculation.

This results in explicit electronic polarization of the buffer region by the I region, effectively reducing artifacts associated with this interface. Panel [B] of Figure 1 illustrates the partitioning of the individual energy contributions of the different regions. It is conceptually useful to decompose the total energy into schematic intra- and inter-region contributions as indicated by the subscripts in eq 2

2 $$V_{\mathrm{tot}}=V_{I,I}^{\mathrm{QM}}+V_{I,B}^{\mathrm{QM}}+V_{B,B}^{QM_{\mathrm{pol}}}+V_{B,B}^{\mathrm{MM}}+V_{B,O}^{\mathrm{MM}}+V_{O,O}^{\mathrm{MM}}+V_{I,O}^{\mathrm{MM}}$$

where the superscripts indicate the level of theory that is used. This decomposition is not intended as a rigorous partitioning of the many-body QM energy, but merely serves to illustrate which interactions are effectively described at the QM and MM levels within the BuRNN framework. The term $V_{B,B}^{{\mathrm{QM}}_{\mathrm{pol}}}$ is defined in eq 3 as

3 $$V_{B,B}^{QM_{\mathrm{pol}}}=V_{B,B}^{\mathrm{QM}}-V_{B,B}^{QM_{\mathrm{isolated}}}$$

where $V_{B,B}^{\mathrm{QM}}$ represents the QM energy within B in the presence of I , while $V_{B,B}^{{\mathrm{QM}}_{\mathrm{isolated}}}$ corresponds to the QM energy of the isolated B , in the absence of I . Consequently, $V_{B,B}^{{\mathrm{QM}}_{\mathrm{pol}}}$ can be interpreted as the polarization of B induced by I . Accordingly, BuRNN can be seen as a localized polarizable embedding strategy, where mutual polarization effects are explicitly retained between I and B . Additionally, O is coupled to all other regions at the MM level. The interactions between O and I can be either embedded mechanically, by using the partial charges of the underlying force field, or dynamically by partial charges of I and B derived from ab initio calculation of I + B . However, this requires an additional partial derivative of the charges with respect to the positions when calculating electrostatic forces between either I or B and O atoms. Within the I + B region, BuRNN goes beyond a purely electrostatic or polarizable description because the learned QM reference energy can include polarization, many-body effects, exchange repulsion, and charge-transfer contributions across the I – B interface. At the same time, the present formulation should not be interpreted as a full polarizable embedding scheme, as the O region does not explicitly enter the electronic Hamiltonian of I + B and is coupled to the remaining regions through MM force-field terms.

The continuity of the buffer region embedding and the relative energetic contributions of buffer-region QM (BuRQM) and MM terms are further analyzed using an explicit hydrogen-bond dissociation scan in Figure S1.

It is shown in Figure 1 that the BuRNN approach requires two QM calculations, which would in practice be exceptionally expensive. Instead, the difference between both QM calculations is directly learned in a MLIP. The difference between $V_{I+B}^{\mathrm{QM}}$ and $V_{B}^{\mathrm{QM}}$ consists of the interaction energy within I , the interaction energy between I and B , and the polarization of B due to I , again described schematically in terms of intra- and inter-region contributions as shown in eq 4

4 $$V_{I+\Delta B}^{\mathrm{MLIP}}=V_{I,I}^{\mathrm{QM}}+V_{I,B}^{\mathrm{QM}}+V_{B,B}^{QM_{\mathrm{pol}}}$$

The term $V_{I+\Delta B}^{\mathrm{MLIP}}$ highlights that the two expensive QM calculations are replaced by the evaluation of a single MLIP. Furthermore, the Δ-sign indicates that BuRNN can be seen as a delta-learning approach for B , where the accuracy of the MM interaction energy is enhanced by QM reference calculations through a correction (delta) energy term to the MM energy.^22^

It is therefore important to note that the BuRNN energy $V_{I+\Delta B}^{\mathrm{MLIP}}$ , which includes both the total energy of I and the interactions between I and B , represents a highly specialized target energy. Consequently, the approach requires dedicated MLIPs that are specifically generated for carefully chosen definitions of I and B . Because the learned target energy depends explicitly on the selected partitioning between I and B , the resulting MLIP is specific to a particular definition of the I and B regions. Consequently, changing the extent of either I or B alters the effective target energy and generally requires retraining of the BuRNN model.

The BuRNN approach was introduced in a proof-of-principle publication using a single Fe­(III) ion in water, placing the coordinative Fe–O bond in hexa-aqua iron at the I – B interface.^19^ This interaction is particularly poorly described by classical force fields, emphasizing the need for a more accurate level of theory. In subsequent work, we introduced a molecular inner region, and performed alchemical free-energy perturbations between methane and methanol in solution, at QM accuracy. In this work we further develop the BuRNN methodology by (i) demonstrating transferability to more complex environments, (ii) standardizing BuRNN for covalent B – O crossings via link-atom strategies and (iii) establishing a robust methodology to generate MLIPs to be used for dedicated BuRNN-tailored MLIPs.

## 2 Methods

In order to expand the complexity of the B four test-systems were prepared. We began with a simple mixture, where interatomic interactions are not the same, resulting in a highly nonideal solution. The next system was chosen as an oversimplified environment of a protein–ligand complex, where we were particularly interested in how the B – O boundary behaves. Next, we return to the Fe­(III) ion and study its interactions within an aqueous heme b system. Here, we focus on how a coordinative bond dissociation is handled. The last case study demonstrates the Cytochrome P450 (CYP450) 1A2 enzyme in its resting state, where the model from the aqueous heme b system is used to describe the interactions within an active site.

All MD simulations were performed using the GROMOS simulation software^23^ and the GROMOS 54A8 force-field parameter set.^24^ GROMOS uses a united atom topology for aliphatic carbons, but to obtain meaningful physical information from the MLIP the explicit information on all atoms are required. Therefore, the implicit hydrogens need to be made explicit, which is done by using the GROMOS++^25^ tool expand_ua to add neutral dummy hydrogens to united-atom centers. The SPC water model was used.^26^ Systems were built in rectangular periodic boxes using GROMOS++ tools. For the charged system (CYP1A2), the simulation box was neutralized with Na^+^ ions, where water molecules were substituted according to solvent positions with the lowest Coulomb potential. An energy minimization step using the deepest descent method was followed by a 100 ps equilibration run in the NVT ensemble at target temperatures *T* = {60, 120, 180, 240, 300} K, each for 20 ps, while loosening position restraint constants on solute atoms by a factor of 10 at each step, from an initial value of 2.5 × 10^4^ kJ mol^–1^nm^–2^. Temperature was controlled during equilibration with a weak coupling thermostat using a relaxation time of 0.1 ps.^27^

Production simulations used a Nosé-Hoover chain thermostat using 5 chains and a relaxation time of 0.1 ps.^28,29^ Separate temperature groups for I , the solute atoms not involving the I , and the solvent were used. Pressure was maintained at 1 atm using a weak coupling barostat with a relaxation time of 0.5 ps and an estimated isothermal compressibility of 4.575 × 10^–4^ (kJ mol^–1^nm^–3^)^−1^.^27^ The time step was set to 0.0005 ps to resolve high frequency modes. The center of mass motion was removed every 1000 steps. Classical electrostatic interactions were calculated using a generalized reaction field with a dielectric permittivity of 61 for aqueous system.^30,31^ A 14 Å cutoff was applied for both electrostatic and Lennard-Jones interactions and the pairlist was generated at every step.

QM calculations were performed with ORCA version 6.0.1.^32,33^ Database entries for the MLIP were labeled using the wB97M-V functional together with the Ahlrichs def2-TZVP basis set.^34–36^ MBIS partial charges were extracted for I + B and the Boys–Bernardi counterpoise correction was applied by introducing ghost atoms having no charge and no mass but contributing basis set orbitals on I atoms when the isolated B calculation is performed.^37–39^

### 2.1 Normal Mode Sampling to Generate Initial Configurations

Initial conformations were generated using a normal mode sampling (NMS) procedure.^2^ Starting from a system of interest, in our case all atoms in I and B regions, a geometry optimization is performed, followed by a frequency calculation using the wB97M-D3BJ functional and a def2-SVP basis set. From the frequency calculation the normal modes *Q* = {*q*_1_, *q*_2_, *q*_3_,...*q*_*N*_*f*__} and their corresponding force constants *K* = {*K*_1_, *K*_2_, *K*_3_,...*K*_*N*_*f*__} are obtained, where *N*_*f*_ is the number of degrees of freedom. Random displacements for each normal mode *i* with *K*_*i*_ > 0 (excluding imaginary modes) are generated according to eq 5

5 $$R_{i}=s_{i}\sqrt{\frac{3c_{i}N_{a}k_{B}T}{K_{i}}},,s_{i}\in \left\{+1,-1\right\},,c_{i}∼U\left(0,1\right)$$

where *k*_B_ *T* is the thermal energy in kJ mol^–1^, *c*_*i*_ a uniform random variate, *N*_*a*_ the number of atoms and *s*_*i*_ a Bernoulli sign with *P* = 0.5. Each displacement is used to scale the normalized normal mode coordinates by *q*_*i*_^*r*^ = *R*_*i*_ *q*_*i*_ and a new conformation is generated by displacing the original coordinates by *Q* ^*r*^ which is the superposition of all *q*_*i*_^*r*^. The energy and forces are calculated from the displaced coordinates. The temperature in eq 5 was set to 2000 K. We emphasize that this NMS protocol is used to generate nonequilibrium configurations to initialize an active learning procedure and it is not intended to sample the canonical distribution at the target temperature.

### 2.2 MLIP Generation and Active Learning

Reference data were generated using an active learning scheme (AL).^40^ In the AL scheme the MLIP is iteratively refined by exploring the PES with short and often biased MD simulations and adding uncertain configurations to the database until a desired accuracy is reached.^10^ A crucial step is to estimate the uncertainty of the predictions, which was calculated in this work using a query-by-committee approach with up to *n*_models_ = 5 independently trained MLIPs, which differed from each other by different training/validation splits.^41,42^ Two uncertainties were calculated during the simulations. The maximum force committee disagreement (σ_F_^max^),^43^ and energy committee disagreement (σ_E_) as in Eqs 6, 7, and 8

6 $$\sigma_{F}^{\max}=\max_{\alpha }\left(\sigma_{F^{\alpha }}\right)$$

7 $$\sigma_{F^{\alpha }}=\sqrt{\frac{1}{n_{\mathrm{models}}}\sum_{i=1}^{n_{\mathrm{models}}}|F_{i}^{\alpha }-\left\langle F^{\alpha }\right\rangle {|}^{2}},,\left\langle F^{\alpha }\right\rangle =\frac{1}{n}\sum_{i=1}^{n_{\mathrm{models}}}F_{i}^{\alpha }$$

8 $$\sigma_{E}=\sqrt{\frac{1}{n_{\mathrm{models}}}\sum_{i=1}^{n_{\mathrm{models}}}|E_{i}-\left\langle E\right\rangle {|}^{2}},,\left\langle E\right\rangle =\frac{1}{n}\sum_{i=1}^{n_{\mathrm{models}}}E^{i}$$

with α as the atom index, hence **F**_*i*_^α^ is the force predicted by the *i*th committee model on atom *α. E*_*i*_ is the energy predicted by the *i*th committee model and ⟨⟩ represent the average predictions. During MD propagations, I+B configurations were extracted with σ_E_ > 5 kJ mol^–1^. As a prequality filter, we performed single-point GFN2-XTB calculations^44^ and applied a maximum force per atom quality filter of 1000 kJ mol^–1^ Å^–1^ to filter out unreasonable structures.

From the remaining pool, *N*_config_ frames are selected with the largest σ_*F*_^max^ to ensure force robustness for MD stability, as reliable force prediction is critical for stable trajectory propagation. Energy-based bounds (−500 to 500 kJ mol^–1^ relative to the reference energy) were enforced before the newly labeled configurations were merged with the existing database, and the Wasserstein distance between the previous and updated energy distributions was computed to quantify distributional shifts. If the Wasserstein distance exceeded 10 kJ mol^–1^, a cold start training was performed; otherwise, training proceeded via warm start. Warm starts reused model weights from the previous AL cycle, with the learning rate reset to 3 × 10^–4^. Cold starts used the default learning rate of 5 × 10^–4^. Warm starts were limited to two consecutive cycles.

Outlier detection was performed using a z-score criterion on the weighted validation scores (80% force, 20% energy) evaluated on the full data set. Committee models with validation scores exceeding 1.5 standard deviations from the committee mean were excluded from the final model selection procedure. No replacement models were trained, such that the effective committee size after filtering could be smaller than the initial maximum of *n*_models_ = 5. After optional removal of outlier models, the final production model was selected as the committee member with the lowest weighted validation score.

To ensure reliability beyond committee disagreement, a ground-truth validation step was triggered if the fraction of configurations exceeding the uncertainty threshold fell below 1% after a minimum cumulative sampling time of 500 ps. In this case, additional evenly spaced configurations (twice *N*_config_) were extracted, labeled to confirm convergence and were added to the database.

MLIPs were trained using SchNetPack 2.0 with the equivariant PaiNN representation using 3 interaction layers, 128 atom-wise features.^45–47^ Interatomic distances were expanded with 20 radial basis functions up to a cutoff of 5 Å. The data set was split into 80% training and 15% validation subsets. We optimized a weighted loss as in eq 9

9 $$L=\omega_{E}{\left(E^{\mathrm{ref}}-E^{\mathrm{pred}}\right)}^{2}+\omega_{F}\frac{1}{n_{\mathrm{atoms}}}\sum_{i=1}^{n_{\mathrm{atoms}}}{∥F_{i}^{\mathrm{ref}}+\frac{\partial E^{\mathrm{pred}}}{\partial R_{i}}∥}^{2}+\omega_{Q}\frac{1}{n_{\mathrm{atoms}}}\sum_{i=1}^{n_{\mathrm{atoms}}}{\left(q_{i}^{\mathrm{ref}}-q_{i}^{\mathrm{pred}}\right)}^{2}$$

with superscripts “ref” and “pred” referring to the reference QM values and the predicted MLIP values, respectively. ω_E_ = 0.05 (kJ mol^–1^)^−1^, ω_F_ = 0.75 (kJ mol^–1^ Å^–1^)^−1^ and ω_Q_ = 0.2 *e* ^–1^, which were optimized to account for the different scales of the properties. Note, however, that the BuRNN simulations in this work did not use fluctuating partial charges for the I and B regions, since the electrostatic forces would require an additional term ∂*q*/∂*r*, which we will address in a future study.

### 2.3 Methanol–Water Mixtures

We simulated a set of five water–methanol mixtures at different methanol mole fractions, *x*_*MeOH*_. The compositions of the production runs are listed in Table 1. The B was defined dynamically as all charge groups within 5 Å of any I atom, updated every time step, where the charge groups were either SPC water molecules or methanol molecules. The BuRNN simulations were setup such that both the methanol and water molecules were treated in separate simulations in the I region, in order to make comparisons to experimental structural data. Furthermore, the reaction-field permittivity for the binary solutions was linearly interpolated from the values used for classical simulations of the pure solutions.^31,48^ The general AL procedure in Figure 2 illustrates the specific approach for the methanol–water mixtures. Methanol composition with I consisting of a water molecule differed from Table 1 at *x*_MeOH_ = 1.00 by having one water in 799 methanols, whereas the methanol composition with I consisting of a methanol molecule differed at *x*_MeOH_ = 0.00 by having one methanol in 1042 waters. During the AL procedure the temperature was increased from 300 to 500 K (at the NVT ensemble, using a weak-coupling thermostat), to bias toward higher energy conformations.^49^ We initially trained models containing a single methanol or water molecule in the I region, and a pure solvent at *x*_MeOH_ = 0.00 and *x*_MeOH_ = 1.00. We performed 12 and 19 rounds of active learning, respectively, consisting of parallel simulations of the central molecule solvated in water and methanol. These rounds of AL led to a combined database consisting of 3580 entries, from which we trained an end point model. The separate end point models were refined by two times ten rounds of active learning (again with either methanol or water in I ), simultaneously running five simulations at different values of *x*_MeOH_ at 300 K. Combining the databases for these refined models, led to a complete model database of 4749 entries, from which the complete model was trained. With this setup we assess the extrapolative power of the MLIP: We compare the behavior of the end point model, which is only trained on the pure solutions, *x*_MeOH_ = 0.00 and *x*_MeOH_ = 1.00 and the complete model, which was refined based on training data with *x*_MeOH_ = 0.00, 0.25, 0.50, 0.75 and 1.00.

**Figure 2:**
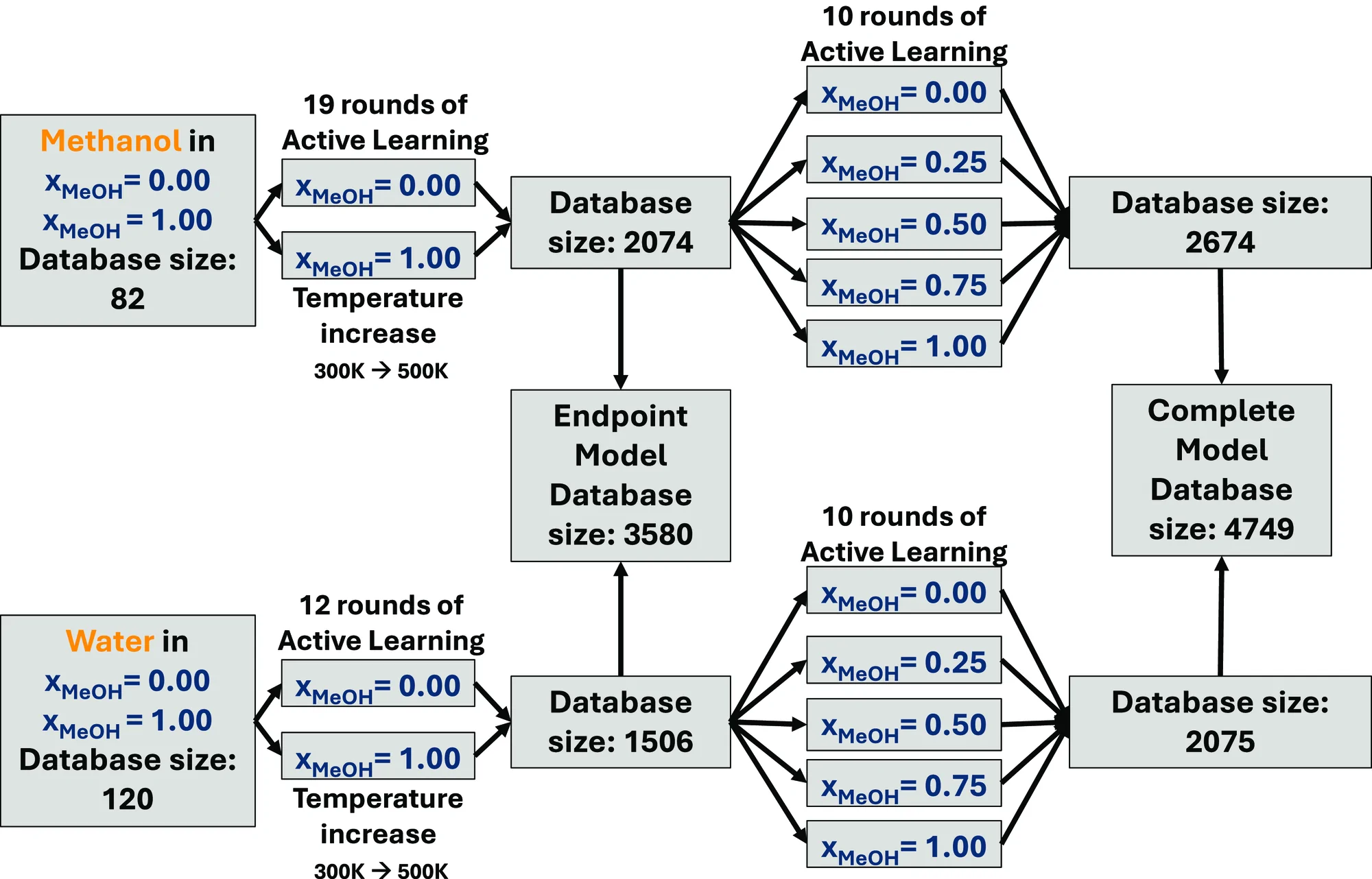
Active learning approach for MLIP generation of methanol–water mixtures. All models contain a single methanol or water molecule in the I region (indicated in orange). Initial models were created using pure solvents, leading to an end point model database of 3580 entries. Refined models, in which the B and O (indicated in blue) contain mixtures were generated, leading to a final database with 4749 entries.

**Table 1:** Methanol Composition in Production MD Simulations with Number of Water and Methanol Molecules, and the Reaction-Field Permittivity Used for Each Simulated System

*x*_MeOH_	*N*_MeOH_	*N*_H_2_O_	ϵ_RF_
0.00	0	1042	61.0
0.20	200	800	53.4
0.25	250	750	51.4
0.50	400	400	41.7
0.75	600	200	32.1
1.00	800	0	22.4

Production runs based on the final models consisted of 10 ns MD simulations at 300 K with a 0.5 fs time step. Bond-length and bond-angle constraints were applied to B and O atoms using the SHAKE algorithm. To analyze the structural behavior of I with its surroundings, the total structure factor *S*(*q*) was constructed. Details of the structure factor calculation are provided in the Supporting Information. To quantify the agreement with experiment, we use the cosine similarity $C_{\mathrm{model}}$ , defined as the normalized inner product of the two curves as in eq 10

10 $$C_{\mathrm{model}}=\frac{\int_{q_{\min}}^{q_{\max}}S_{\mathrm{ref}}\left(q\right)S_{\mathrm{model}}\left(q\right)dq}{\sqrt{\int_{q_{\min}}^{q_{\max}}S_{\mathrm{ref}}{\left(q\right)}^{2}dq}\sqrt{\int_{q_{\min}}^{q_{\max}}S_{\mathrm{model}}{\left(q\right)}^{2}dq}}$$

Here, *S*_ref_ denotes the experimental structure factor and *S*_model_ the corresponding structure factor obtained from either the MM or BuRNN model. The cosine similarity measures agreement in the overall shape of the curves and is bounded between −1 and 1, with $C_{\mathrm{model}}=1$ corresponding to perfect agreement. We evaluate the structure factor *S*_model_ on the experimental *q*-grid in the domain $D=\left\{q|q_{\min}\leq q\leq q_{\max}\right\}$ with *q*_min_ = 1 Å^–1^ and *q*_max_ = 10 Å^–1^. The lower bound excludes the low-*q* region where finite-size effects dominate the behavior. Model curves are interpolated onto the experimental grid. The experimental structural data was collected from X-ray diffraction (XRD) data at room temperature.^50^ Except for *x*_MeOH_ = 0.25, data is available for all simulated molar fractions. The simulations at *x*_MeOH_ = 0.25 are compared to the next closest experimental mole fraction of *x*_MeOH_ = 0.2.

### 2.4 Artificial Buckyball

A C_60_-like fullerene functionalized with a carboxylic acid that traps a methanol molecule was constructed, emulating a minimal enzyme active site (see Figure 3).^51^ Methanol comprised the I region and the carboxylic acid the B region, hence covalently crossing the B – O boundary. The optimal C–C bond lengths in the fullerene were artificially set to 2.00 Å, except for the carboxyl linkage C–C_carboxy_ which was set to 1.53 Å. Furthermore, the fullerene was simulated in an open form. The production runs consist of a 200 ps MD simulation in vacuum with 0.5 fs step at 300 K, while keeping all bonds unconstrained.

**Figure 3:**
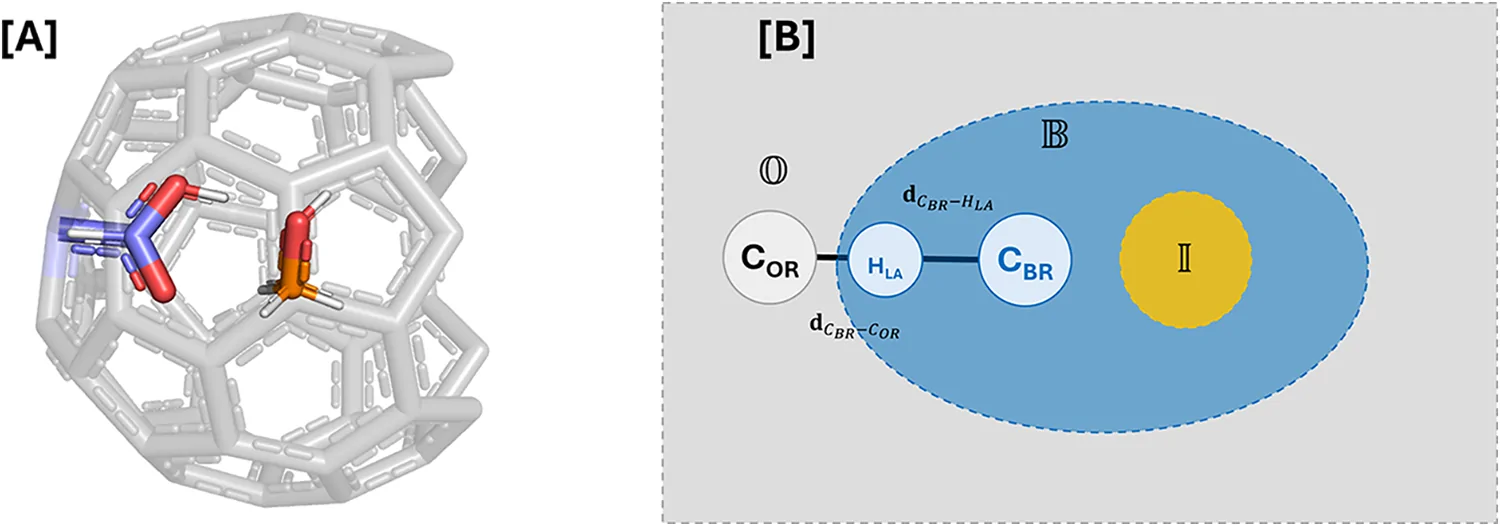
Representation of Buckyball model. Panel [A] highlights the methanol and carboxyl group covalently bound within the fullerene, which is shown transparently to improve visibility. Panel [B] highlights the interface and the treatment of the B – O interface via an explicit link atom.

The simplest and easiest solution to the problem of cutting covalent bonds in QM/MM is the circumvention of it by defining the subsystem such that the boundary does not pass through a covalent bond. While this is trivial for liquids it becomes nontrivial for macromolecules such as proteins, where active-site residues often cross boundaries. Classical QM/MM approaches utilize methods such as link atoms (LA).^15^ Here additional atomic centers, typically hydrogens, are introduced on the QM region and occasionally on the MM region to cap the bond and satisfy the free valency. In BuRNN, the B region interacts with the O region at the MM level, eliminating the need to define a capping for the MM part. We evaluated two training strategies for handling the B – O bond on the QM side.

#### 2.4.1 Approach 1: Training on Physical Molecules—Explicit Link Atom Scheme

A hydrogen LA was placed along the C–C_carboxy_ bond, forming H_LA_–C_carboxy_ in the QM reference calculation. The BuRNN model was trained on these physical molecules, including the LA coordinates, energy, forces and partial charges. During BuRNN simulations, the same LA was introduced and the force acting on the link atom was redistributed to the boundary atoms (C_carboxy_) in B and C in O . This approach is illustrated in panel [B] of Figure 3.

#### 2.4.2 Approach 2: Training on Non-Physical Molecules—Implicit Link Atom Scheme

Here, the QM calculation was performed as in Approach 1, but prior to training the coordinates, forces and partial charges of the LA were removed, while the QM energy was left unchanged. The model hence learns the full energies from implicitly capped (unphysical) molecules. This creates a temporary inconsistency between energy and forces in the training targets, introducing noise, that the model must reconcile. However, during training, the model learns how to distribute the missing cap LA forces according to the learned representation, since forces are obtained analytically as **F** = −∇*E*, such that energy and forces are consistent at the inference.

In both approaches, errors due to capping are made twice and largely cancel in the difference between the two QM calculations in Figure 1. Furthermore, the remaining error is localized rather far away from I , supporting the approximation that the interaction between the link atom used in the capping and the I molecule may be negligible.

Additionally, we performed QM/MM simulations with mechanical embedding as comparison, where the QM region comprised methanol and the carboxyl group with the same LA definition as in the BuRNN approach. This was done using the semiempirical tight-binding model GFN2-XTB.

### 2.5 Aqueous Heme b

To extend the complexity in the B and to follow up on the first BuRNN paper, where an Fe­(III) was simulated in aqueous solution, we constructed a heme b system, where four porphyrin nitrogen atoms account for the equatorial coordination and a cysteine serves as the proximal axial ligand. The cysteine backbone was acetylated at the N-terminus and capped with an *N*-methyl group at the C-terminus to yield a neutral backbone. SPC water will take up the distal axial ligand of the iron in the solvated state. The I region comprised Fe­(III), whereas the B region statically included the porphyrin macrocycle and axial thiolate, and dynamically all water molecules within 5 Å of I . A representational structure is shown in Figure S4. This setup introduces nine link atoms (8 on the porphyrin and one on the thiolate). The heme b model mimics cytochrome P450 enzymes in their resting state, which typically exhibit a low-spin (doublet) spin state. Upon binding of a substrate to the active site of the enzyme, the coordinating water molecule is displaced and a five-coordinated high-spin (sextet) ferric complex is formed. Here, we focus on the low-spin potential energy surface and demonstrate that BuRNN can describe dissociation of the distal water ligand. Extension to multiple electronic states will be addressed in future work.

#### 2.5.1 Umbrella Sampling of Dissociation of Water Molecule from Fe­(III) in Heme B

In order to simulate dissociation of the water molecule from Fe­(III), a reaction coordinate needs to be defined. We chose the coordination number of the ferric ion with respect to all water oxygen atoms in the simulation box ξ_Fe–O_W__,^52^ which is defined as in eq 11

11 $$\xi_{\mathrm{Fe}-O_{W}}=\sum_{i}^{N_{O_{W}}}\frac{1-{\left(\frac{r_{\mathrm{Fe}-i}}{R_{0}}\right)}^{\mathrm{NN}}}{1-{\left(\frac{r_{\mathrm{Fe}-i}}{R_{0}}\right)}^{\mathrm{ND}}}$$

where *r*_Fe–*i*_ is the distance between the iron atom and the oxygen atom *i* of any water molecule, *R*_0_ is the distance cutoff, which is set to 3.0 Å, *N*_O_W__ is the number of water molecules in the simulation box and the exponents *NN* and *ND* determine the steepness of the step function with values of 20 and 40, respectively. A harmonic biasing potential was used to explore the free-energy landscape along the reaction coordinate as in eq 12

12 $$V_{\mathrm{bias}}\left(\xi \right)=\frac{k}{2}{\left(\xi_{\mathrm{Fe}-O_{W}}-\xi_{0}\right)}^{2}$$

where ξ_0_ defines the target coordination number and *k* the associated force constant. An initial model was constructed from 186 NMS configurations generated at 2.1 ξ_Fe–O_W__ = 0 and ξ_Fe–O_W__ = 1 and a temperature of 2000 K. Active learning was conducted over 18 iterations using eight parallel umbrella windows spanning different ξ_Fe–O_W__ values (force constants listed in Table S2). In total, 144 short biased MD simulations were performed during the AL procedure. The umbrella potentials were not required by the AL methodology itself, but were introduced to ensure sufficient sampling along the water dissociation pathway. Since spontaneous water dissociation events are rare on accessible MD time scales, unbiased simulations would not adequately sample the relevant high-energy regions of the PES.

Configurations exhibiting spin contamination exceeding 0.1 relative to the expected ⟨*S* ^2^⟩ value were discarded. In total, 971 configurations were added during active learning, resulting in a final training set of 1157 structures. Production umbrella sampling simulations were then carried out at 21 values of ξ_Fe–O_W__ (Table S3). Each window was equilibrated for 20 ps, followed by a 1 ns production run at 300 K using a 0.5 fs time step, with all bonds unconstrained. The final configuration of each equilibration served as the starting structure for the subsequent window. Free-energy profiles were obtained using an iterative WHAM procedure; further technical details are provided in the Supporting Information.

### 2.6 Cytochrome P450 1A2

To transfer the heme b into a protein environment, we selected cytochrome P450 1A2 (CYP1A2) because its binding pocket is comparatively rigid and water-filled in the resting ferric state of the catalytic cycle,^53^ making it well suited to transfer the MLIP trained for the solvated heme b system. The same I + B composition and MLIP was used as for the solvated heme b and is illustrated in Figure S4 with the protein environment present. An unbiased MD production run of 1 ns at 300 K with a 0.5 fs time step was performed to characterize the resting state of CYP1A2, while keeping only the conformation of SPC molecules constrained via SHAKE. Trajectories were analyzed to assess the stability of the metal coordination and the stability of a BuRNN simulation in a protein environment.

## 3 Results and Discussion

### 3.1 Methanol—Water Mixtures

The active-learning end point and complete models were used to perform stable 10 ns MD simulations using BuRNN. While a BuRNN simulation is approximately 40% slower than an MM simulation, it is almost 300 times faster than a QM/MM simulation with a single methanol molecular in the inner region and 30,000 times faster than a BuRQM simulation, requiring two larger QM calculations. See Table S1 in the Supporting Information for further information.

To assess the quality of the MLIPs and the BuRNN approach, we validated the water–methanol models against experimentally measured total X-ray structure factors. The X-ray structure factor for the mixtures were constructed from separate BuRNN simulations, in which either a methanol or a water molecule were included in the I , enabling a fair comparison with the experimental data.

Figure 4 compares the BuRNN complete model and the MM reference (simulated with the same settings as BuRNN simulations) to experiment. We note that the structure factors are limited by the poor statistics of the radial distribution functions (RDFs) at longer distances which would take longer to fully converge. The lack of convergence is seen in the region *q* < 1 Å^–1^ for both the BuRNN and the MM simulations. The BuRNN model shows closer agreement in the first peak around *q* = 2 Å^–1^, whereas the second peak at around *q* = 3 Å^–1^ is overall comparable between BuRNN and MM. In previous work, discrepancies in these two peaks were observed for different force fields,^54^ which are commonly interpreted as inconsistensies in the intermolecular interactions of a highly nonideal mixture. It is reassuring to observe that the BuRNN simulations match, or even outperform classical force field models in reproducing these peaks.^55^ At higher values of *q*, BuRNN consistently reproduces the oscillatory pattern of the experimental curves more accurately than MM. The overall higher similarity scores of BuRNN relative to experimental data highlight that first a MLIP/MM scheme improves the description of intramolecular interactions and accurately reproduces the anharmonicity from the QM reference. Second, the buffer region embedding enhances the treatment of intermolecular interactions between the I and the O regions as reflected in the improved agreement in the first peak.

**Figure 4:**
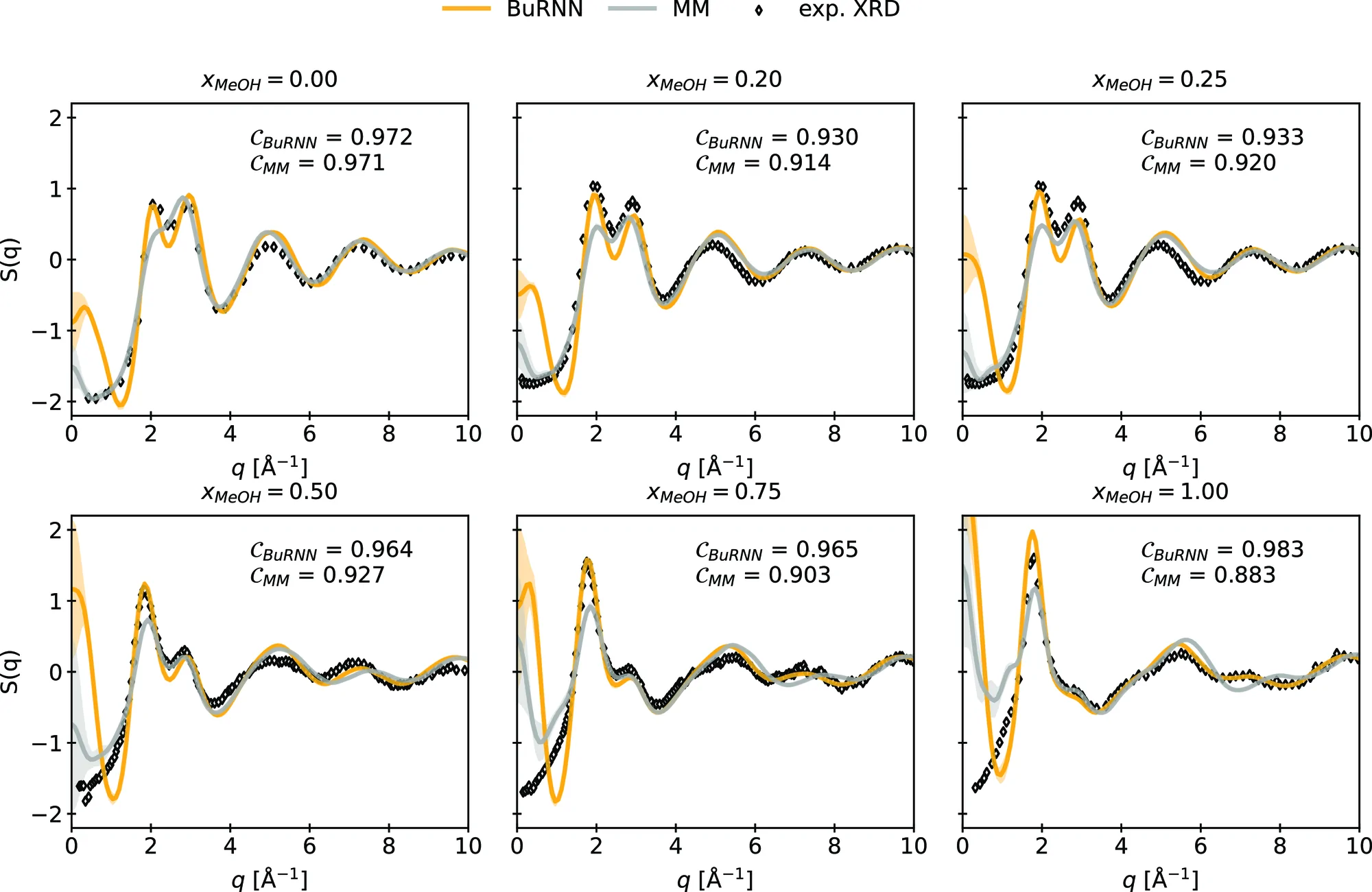
Structure factors for water—methanol mixtures at compositions *x*_MeOH_ = 0.00, 0.20, 0.25, 0.50, 0.75, 1.00. Experimental data are shown in black-edged markers, Structure factors computed from BuRNN simulations as orange lines, and those from MM simulations as gray lines. Shaded bands represent statistical uncertainty estimated via block averaging by dividing the simulation into four blocks. Note that for *x*_MeOH_ = 0.25 no experimental data was available, but the data of *x*_MeOH_ = 0.20 is shown. Additionally, the similarity score evaluated on the experimental grid within the specified scoring window (eq 10) is shown for each composition.

To further assess the structural behavior at longer distances and to investigate whether the deviations in the low-*q* region originate from inconsistencies at the B – O boundary, the corresponding oxygen–oxygen RDFs are reported in the Supporting Information (Figure S2). The RDFs explicitly show the statistical uncertainty estimated from block averaging and indicate that the long-range oscillations are only partially converged for some compositions, consistent with the increased uncertainty observed in the structure factors at low *q*. Importantly, no discontinuity or pronounced artifact is observed around the B cutoff, suggesting that the deviations in the low-*q* region do not arise from inconsistencies between the B and O regions.

Additionally, vibrational power spectra calculated from the BuRNN simulations are shown in Figure S3. The spectra show composition-dependent shifts, providing further evidence that the BuRNN approach captures the local environmental effects and anharmonic interactions relevant for condensed-phase dynamics.

Furthermore, we investigated how a BuRNN end point model performs for structural properties in mixed compositions by running stable production simulations based on the end point model and, alongside simulations based on the complete model from Figure 4, compare them to the experimental data. Since the end point model and the complete model generate distinct trajectories, the resulting similarity scores (Eq 10) reflect both model difference and dynamic sampling differences. The similarity scores in Table 2 show that the end point model reproduces the total structure factor nearly as well as the complete model across compositions, indicating that the overall structural behavior of mixtures is already captured by the end point model despite its limited training domain.

**Table 2:** Cosine Similarity Scores Comparing BuRNN Predictions to Experimental Data for Methanol-Water Mixtures^a^

*x*_MeOH_	$C_{\mathrm{BuRNN}}^{\mathrm{EM}}$	$C_{\mathrm{BuRNN}}^{\mathrm{CM}}$
0.00	0.980	0.972
0.25	0.931	0.933
0.50	0.966	0.964
0.75	0.951	0.965
1.00	0.971	0.983

a Two BuRNN variants are reported: the end point model (EM, trained on data at *x*_MeOH_ = 0.00 and) *x*_MeOH_ = 1.00 and the complete model (CM, trained on all compositions).

To further assess the reliability of the end point model, we compared the errors of the production run at a molar fraction *x*_MeOH_ = 0.50 for both the complete and end point models. For this purpose DFT energies and forces were recalculated for 200 configurations, extracted every 100 ps, with either methanol or water present in I . The results are shown in Figure 5. The end point model shows larger errors in both quantitities, consistent with its training only on the pure end points. Nevertheless, the error distributions of this model remain within a reasonable range for mixed compositions. The mean-absolute error (MAE) and root-mean-square error (RMSE) in the energy deviate only modestly from our target accuracy of 1–2 kJ mol^–1^ for production simulations.

**Figure 5:**
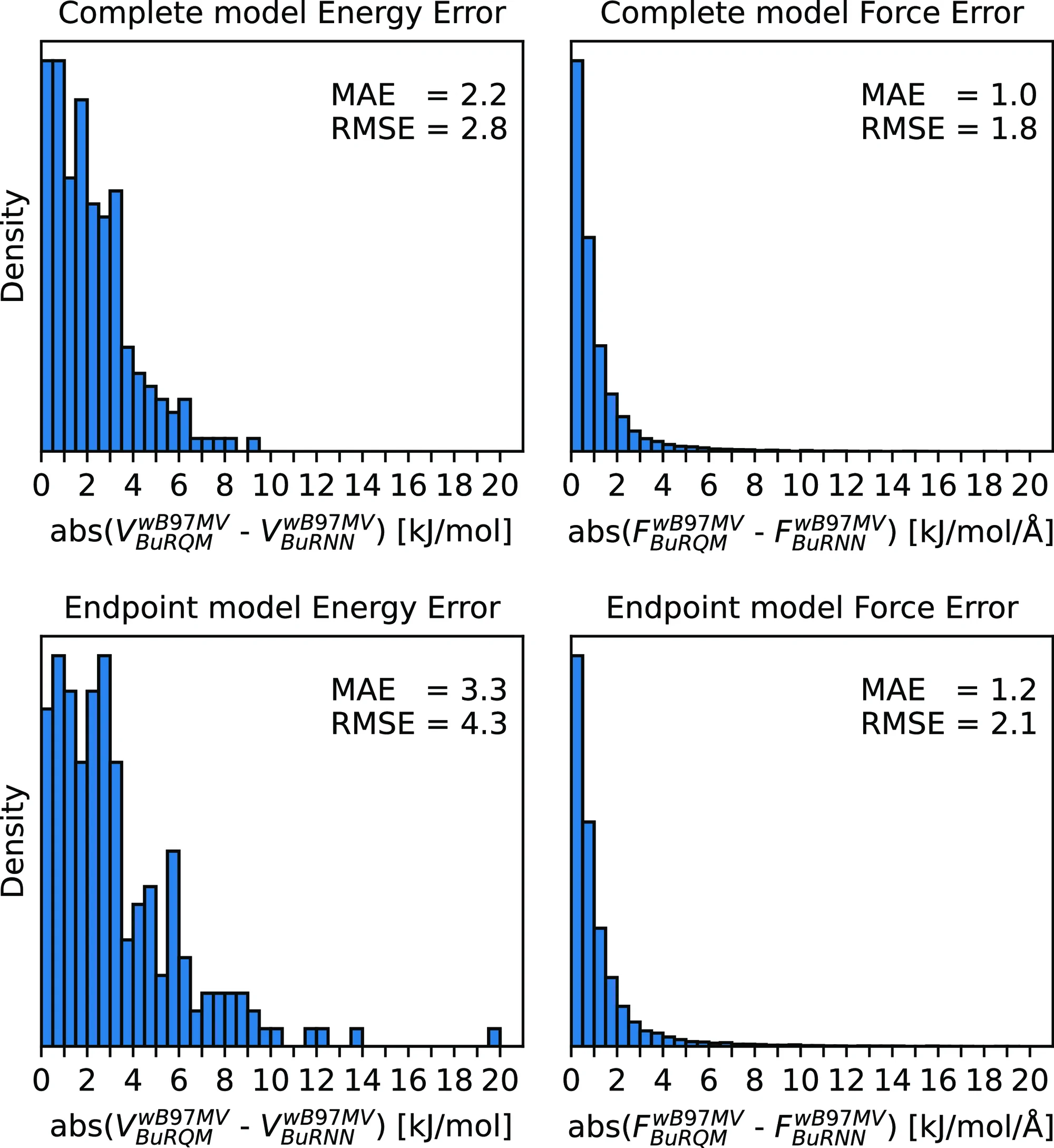
Error comparison of production runs at molar fraction *x*_MeOH_ = 0.50. Histograms show absolute errors with respect to the recalculated energies and forces using the DFT wB97M-V ground truth for 200 configurations extracted from the BuRNN simulations. The top row reports the complete model, and the bottom row the end point model. Left panels display errors in the energy, where right panels display errors in the forces. Insets list the mean-absolute error (MAE) and the root-mean-square error (RMSE) for each panel.

### 3.2 Artificial Buckyball

We compare three diagnostics to quantify the quality of the description of the bond across the B – O boundary: (i) Energy committee disagreement according to eq 8; (ii) variation in the bond length of the bond crossing the B – O boundary; and (iii) magnitude of the torque on the link atom (LA). The first diagnostic is displayed in panel [A] of Figure 6 for both the explicit and implicit LA scheme. The implicit LA scheme produces a considerably broader distribution of the energy committee disagreement compared to the explicit LA approach. In a protein, where multiple covalent crossings may occur this effect will be expected to be even more pronounced. The B – O bond length in the explicit LA approach, exhibits two atoms of a bond getting additional forces from the LA. Panel [B] shows that, relative to a classical MM simulation of the same system, the bond-length distribution is identical, indicating no artificial distortion at the B – O boundary. In the implicit LA approach, the bond is purely described by MM, leading to a very similar distribution as well (data not shown).

**Figure 6:**
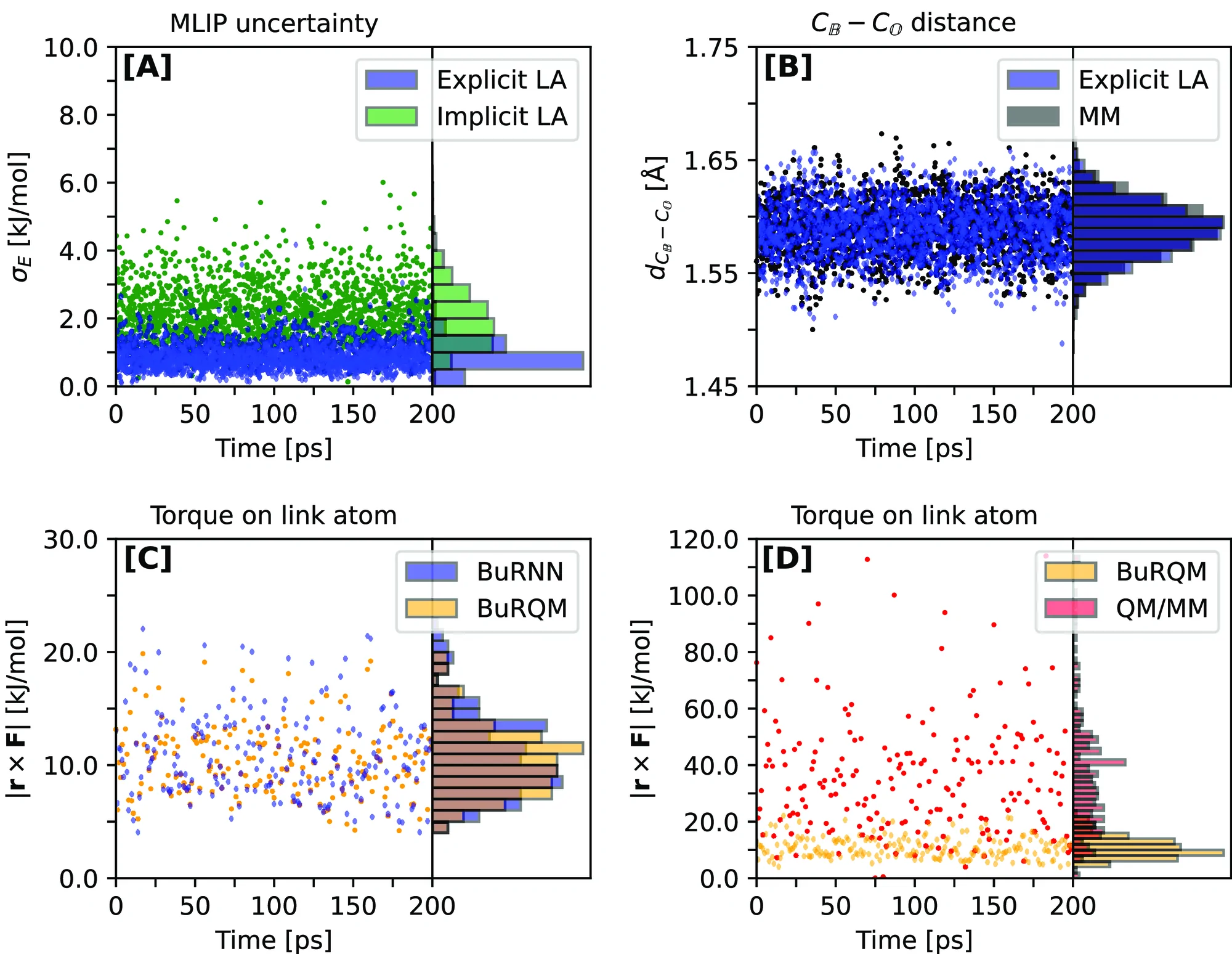
Buckyball link atom boundary analysis. [A] Time series and distribution of the energy committee disagreement for the explicit and implicit LA schemes. [B] Time series and distribution of the B – O boundary bond length for the explicit LA scheme and a classical MM simulation as reference. [C] Time series and distribution of the magnitude of the torque on the LA in BuRNN and as recalculated using QM calculations (BuRQM). [D] Time series and distribution of the magnitude of the torque on the LA in QM/MM and as recalculated in the BuRQM approach.

We analyze the magnitude of the torque on the LA to characterize the forces along the boundary. The torque was computed *a posteriori* per frame by forming the bond vector H_LA_–C_carboxy_ and taking its vector product with the force acting on the LA. Panel [C] shows the LA torque time series and the distribution from BuRNN together with torques recomputed at the reference level (explicitly performing the two QM calculations of Figure 1; BuRQM) for the same frames. The close agreement between BuRNN and BuRQM indicates that the observed torque is intrinsic to the LA boundary itself rather than an artifact of MLIP uncertainty. Note that in an implicit LA approach, this torque cannot be accurately represented, which is likely the cause of increased variation between models observed in panel [A]. Lastly, panel [D] compares BuRQM to a QM/MM mechanical-embedding calculation with the same LA definition. The QM/MM torque magnitude on the LA using mechanical embedding is approximately 4-fold larger than in BuRNN. This can be explained from the fact that the LA in BuRNN reflects only the interaction with the I region, whereas in QM/MM the LA bears the full hydrogen force. In other words, in BuRQM (and BuRNN), the force on the LA largely cancels in the energy difference, and only the contribution of I on the LA needs to be learned.

Overall, the explicit LA training delivers lower uncertainty and a more precise B – O boundary behavior than the implicit LA training. In larger biomolecular systems with multiple covalent crossings the uncertainty penalty of the implicit LA strategy is expected to add even more noise, hence making the explicit LA approach the preferred choice.

### 3.3 Aqueous Heme b

The structural integrity of the solvated heme b model in aqueous solution was assessed by analyzing key geometric parameters, such as the Fe-ligand bond-lengths and the out-of-plane position of the iron in the porphyrin ring as shown (Figure 7) for the resting state with ξ_Fe–O_W__ = 1. The distributions of the bond distance from the BuRNN simulation show a stable coordination. The Fe–N distances showed a narrow distribution with a mean at 1.988 Å, which is in good agreement with X-ray data of 2.008 Å.^56^ Similarly, the out-of-plane position of the Fe was measured by calculating the average improper dihedral, defined by the nitrogens of the porphyrin. BuRNN captures a slight tendency of the resting state to be in a out-of-plane position. The simulated average Fe–S (2.212 Å) and Fe–O (2.166 Å) distances are both shorter, compared to the experimental X-ray structure of 2.25 Å and 2.28 Å, respectively. To facilitate a visual interpretation, a ± 0.2 Å comparison window is shown in Figure 7. This range provides a conservative visual tolerance for agreement. For macromolecular structures refined at 1.6 Å resolution, coordinate uncertainties are typically on the order of 0.1 Å or smaller,^57^ such that the applied window represents a conservative bound rather than a strict experimental error estimate. It has been shown in the past that deviations of these distances compared to X-ray structures in DFT/MM studies occur.^58^ Hence, an additional comparison was done on a geometry optimized structure with the same underlying functional and basis set, which was performed on the I + B regions. The mean of the Fe–S distance distribution matches the minimized data (2.208 Å), as is the case for the Fe–O distance (2.064 Å).

**Figure 7:**
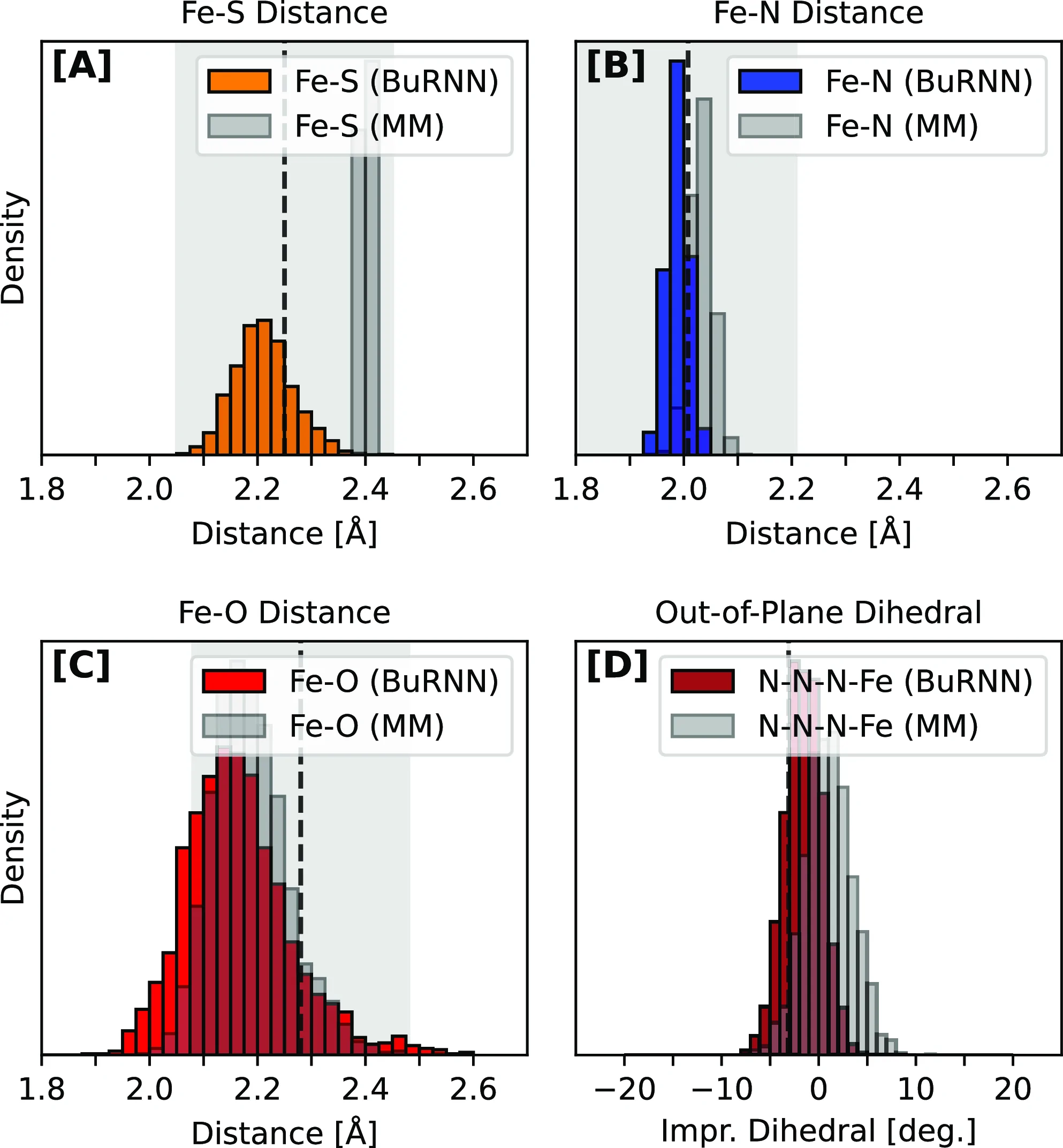
Comparison of geometric heme b parameters obtained from BuRNN and reference MM simulations at ξ_Fe–O_W__ = 1 in aqueous solution. Distribution of [A] Fe–S bond distance, [B] mean of Fe–N­(porphyrin) distances, [C] Fe–O distance of the closest water molecule, and [D] mean of out-of-plane porphyrin N–N–N-Fe improper dihedral angles. BuRNN results are shown in color, while MM reference data are shown in gray. The vertical dashed lines indicate experimental values derived from the crystal structure (PDB ID: 1PHC). Gray shaded regions in panels A–C denote a ± 0.2 Å comparison window around the experimental bond distances to guide visual assessment of agreement.

Figure 8 shows the potentials of mean force (PMFs) for water dissociation along the reaction coordinate obtained by the weighted histogram analysis method. Time series and histograms of ξ_Fe–O_W__ from the 21 individual simulations can be found in Figure S6. The BuRNN and MM PMFs exhibit, in general, similar qualitative profiles as can be seen by the minimum at ξ_Fe–O_W__ = 1 and the increment toward lower coordination values due to the weaker interaction of Fe–O and an artificial, but small vacuum which is formed at the distal site. Quantitatively, the BuRNN curve shows a weaker interaction of the water molecule to the Fe­(III), reflected in the smaller barrier height. For the pentacoordinated state at ξ_Fe–O_W__ = 0 the same geometric analysis as in Figure 7 is shown in Figure S5 in the Supporting Information. The average Fe–N distance becomes slightly shorter upon water dissociation from 1.998 Å to 1.964 Å, whereas they should become larger from 2.008 Å to 2.095 Å. Additionally, the Fe–S distance is underestimated in the dissociated state by approximately 0.191 Å. On the other hand, the out-of-plane position becomes more pronounced, which is in line with the experimental data.^59^ We note that quantitative comparison to experimental properties for the pentacoordinated iron requires the explicit treatment of alternative electronic ground (sextet and quartet) for the ferric complex. The present results pertain to the doublet PES used for training and sampling and demonstrate the feasibility of performing bond dissociation simulations in metal–ligand bonds.

**Figure 8:**
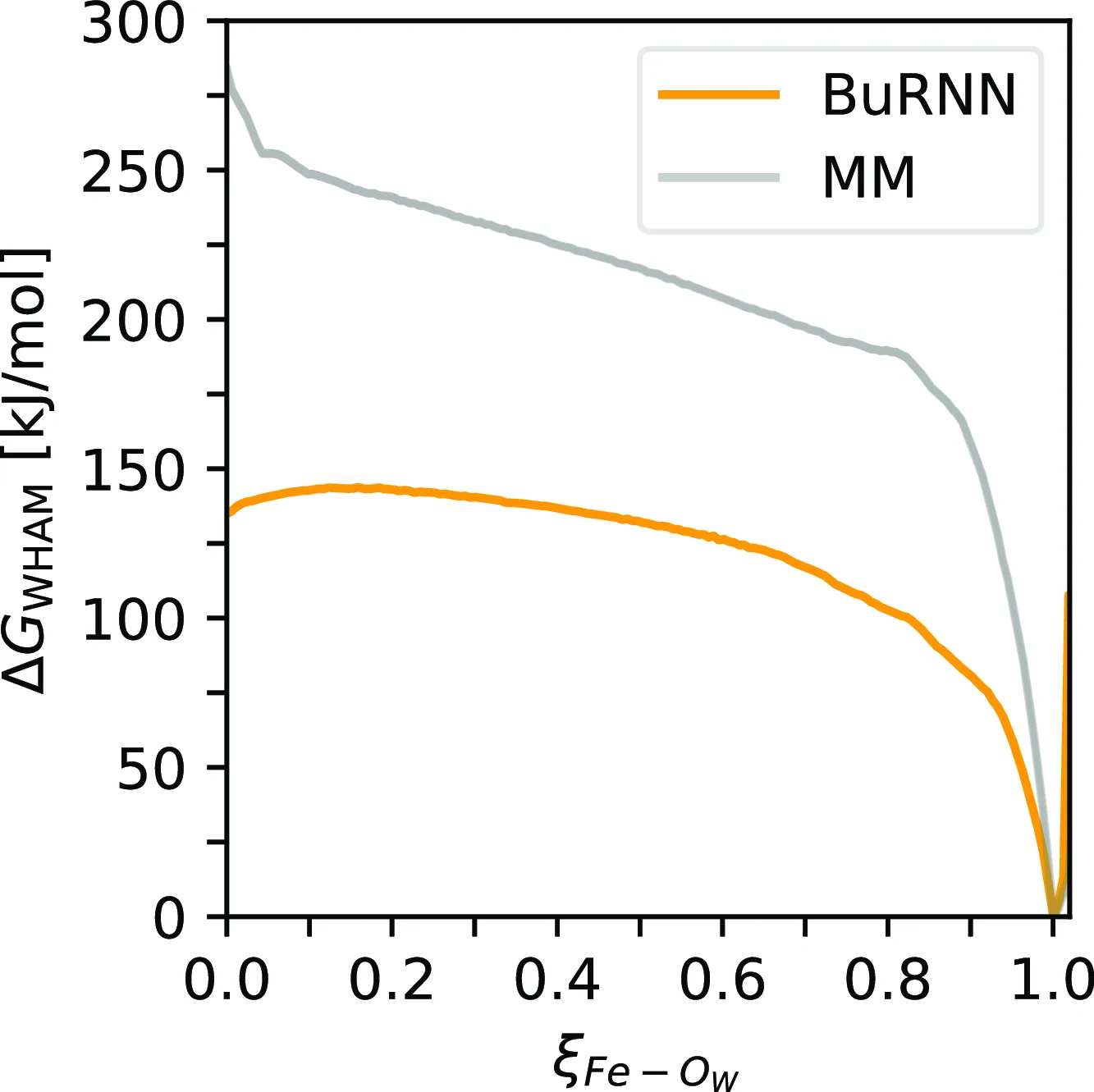
Potential of mean force reconstructed from the biased umbrella sampling bins using the weighted histogram analysis method (WHAM) as a function of the iron-water coordination number. Orange shows the BuRNN results and black shows the MM results.

### 3.4 Cytochrome P450 1A2

The structural integrity of the heme b model, embedded in the CYP1A2 enzyme was assessed by analyzing the same geometric parameters, as in Figure 7. The Fe–N and Fe–S bond lengths and the out-of-plane position of the iron display the same distribution as in the aqueous environment, whereas the Fe–O distance gives rise to a narrower distribution as is shown in Figure S7.

The effect of BuRNN in the protein environment was further assessed by analyzing the orientation of the distal axial water molecule in Figure 9. Time series and probability distributions of two orientational descriptors are reported: (i) The tilt angle, defined as the angle between the water dipole bisector and the Fe–O bond vector, and (ii) the normal angle, defined as the angle between the normal vector of the H–O–H molecular plane and the Fe–O bond vector.

**Figure 9:**
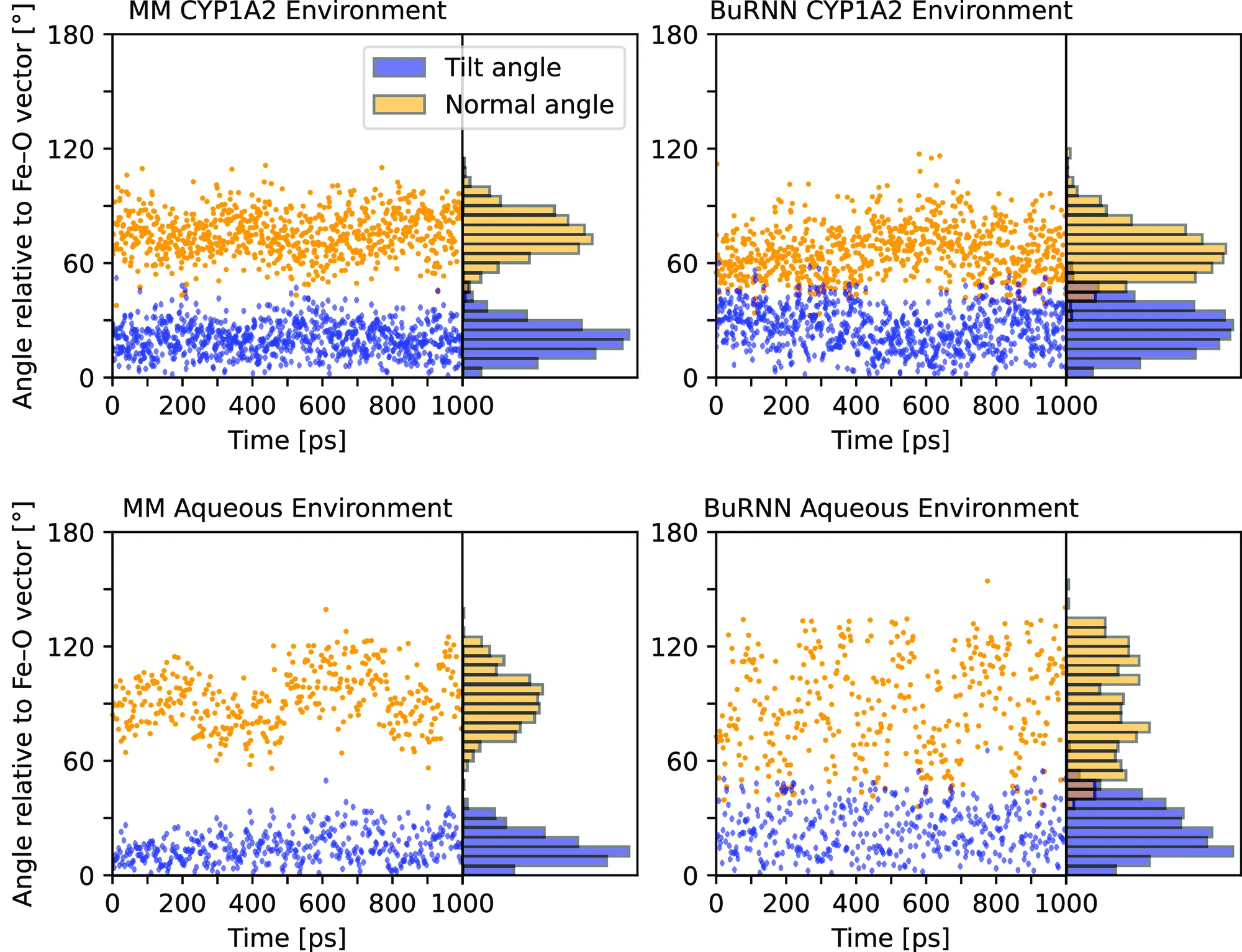
Time series and probability distribution of the orientation of the distal axial water molecule at ξ_Fe–O_W__ = 1. The left column displays the result using MM, whereas the right column displays the result using BuRNN. The first row corresponds to the CYP1A2 simulations, whereas the second row displays the aqueous environment. Two properties are shown, the tilt angle (blue), and the normal angle (orange).

In the aqueous environment, the tilt angle has a mean of 24° and 15°, for BuRNN and MM respectively, indicating a preferential alignment of the water dipole toward the iron center. This suggests that the axial water consistently adopts a bent axial coordination geometry. The tilt angle for BuRNN is slightly larger than in MM, as the coordinative bond will involve one of the lone-pair orbitals of the oxygen atom. In MM, no electronic effects are reflected, leading to smaller tilt angles. The normal angle has a mean of 89° and 92°, for BuRNN and MM, where BuRNN simulations show a wide distribution spanning roughly 30–150°, indicating a substantial rotational freedom of the water molecule around the Fe–O bond. Thus, while the water dipole maintains a preferred tilt relative to the metal center, the H–O–H plane is able to sample a wide range of orientations. In the CYP1A2 simulations, the tilt angle has a mean of 25° and 20°, for BuRNN and MM respectively, very comparable to the values in aqueous solution. In contrast, within the CYP1A2 active site, the normal-angle distribution becomes noticeably narrower and shifts to a mean of 67°, with a reduced range of approximately 30–100°. This narrowing indicates a loss of rotational freedom of the axial water molecule, likely due to specific interactions in the distal pocket that bias the orientation of the H–O–H plane while preserving the preferred dipole alignment toward the iron. Overall, these results indicate that the protein environment significantly influences the rotational orientation of the coordinated water molecule, whereas the metal–water bonding geometry, as reflected by the tilt angle, remains largely conserved.

Additional analyses of the protein and the active site environment are provided in Figure S8. The active-site hydrogen-bond occupancies are very similar between the MM and BuRNN simulations indicating that the local hydrogen-bonding network is preserved. Likewise, the distal pocket water occupancy distributions are comparable, but within the 0.5 nm B cutoff used in the BuRNN simulation, BuRNN consistently has three water molecules present, whereas the classical MM simulation retains a small probability of configurations with two water molecules. The DSSP analysis^60^ further confirms that the secondary structure remains stable over the simulation.

## 4 Conclusion

A comprehensive study was conducted to expand the usage of the buffer-region embedding for MLIP/MM simulations in complex environments. We studied the possibilities of interpolation and transferability of BuRNN models, and compared two recipes to handle bonds across the B – O boundary. Across liquid mixtures, aqueous heme b, and a protein active site, the approach reproduced experimental observables or the underlying QM reference data, hence making it suitable for complex condensed-phase systems. We explored alternative capping schemes and found that the explicit treatment of LA provided a more robust behavior in the estimation of uncertainties, compared to the implicit capping. The uncertainty in the models are of critical relevance when performing an active learning procedure to generate a MLIP. In the methanol–water mixtures, models trained on end points (pure-composition data) generalized well to mixtures, and B -mediated interactions preserved experimental structural features at a similar accuracy as on a model trained on intermediate compositions. This transferability to unseen B compositions serves as a first step to create generalized models, which are able to dynamically describe interactions within a protein.

While BuRNN was initially validated for a hexaaqua iron complex in water, we extended the approach toward biologically relevant iron interactions as they are present in heme proteins. We showed that BuRNN can produce stable simulations of heme b during coordinative bond dissociation, while preserving good agreement with the underlying QM reference data and experimental observations. Since systems involving transition-metals often possess a dense orbital manifold and closely lying spin and electronic states, this opens the opportunity for future work on (i) incorporating fluctuating-charges to accurately capture long-range, conformation- and composition-dependent polarization, effectively allowing charge transfer between I and B , and (ii) electronic embedding that treats multiple spin and electronic states explicitly, enabling quantitative comparisons for pentacoordinate intermediates and spin-state dependent reactivity.

## Supplementary Material



## Data Availability

Trained models, input parameters, topologies, and molecular configurations are available at: https://doi.org/10.5281/zenodo.19207122. The GROMOS program and the package to run the active learning pipeline are available at: https://github.com/mms-boku/BuRNNpack and https://github.com/biomos/gromosXX.

## Notes

Additional Information on the structure factor calculation. Radial distribution functions, power spectra of the methanol–water mixtures, solvated heme b geometry distributions, umbrella sampling parameters, WHAM analysis, bootstrap uncertainty estimation, and structural analysis for CYP1A2 (PDF)
