# Supplementary material for: Buffer Region Embedding for Hybrid Machine-Learned/Molecular-Mechanical Simulations in Complex Environments
Source: J Chem Inf Model. 2026 Aug 4;66(16):10019–32. doi: 10.1021/acs.jcim.6c00924 (PMC13508784; doi:10.1021/acs.jcim.6c00924)
Supplement: Supplementary file 1 [file ci6c00924_si_001.pdf]

Supporting Information: Buffer Region Embedding for Hybrid Machine-Learned / Molecular-Mechanical Simulations in Complex Environments

Michael Caspary,[†,‡] Radek Crha,[†,‡] Edgar Galicia-Andrés,[†,‡] Peter Poliak,[†,¶] and Chris Oostenbrink*,[†,‡]

†*Institute for Molecular Modeling and Simulation, BOKU University, 1190 Vienna, Austria*

‡*Christian Doppler Laboratory for Molecular Informatics in the Biosciences, BOKU University, 1190 Vienna, Austria*

¶*Institute of Physical Chemistry and Chemical Physics, Faculty of Chemical and Food Technology, Slovak University of Technology in Bratislava, Radlinského 9, Bratislava 812 37, Slovakia*

E-mail: chris.oostenbrink@boku.ac.at

Contents

Buffer Region Embedding: Additional Information S-2

Methanol-Water Mixtures: Additional Information S-4

Solvated heme b: Geometry distributions and umbrella sampling S-9

CYP1A2: Additional Analysis S-14

Buffer Region Embedding: Additional Information

To further illustrate the behavior of the buffer-region embedding scheme, we computed the potential energy surface associated with the dissociation of a hydrogen bond between an isolated methanol–water pair in vacuum. During the scan, the methanol hydroxyl hydrogen to water oxygen distance was increased from 1 Å to 7 Å in 61 consecutive rigid displacement steps, while all remaining internal coordinates were kept fixed.

The methanol molecule was assigned to the $\mathbb{I}$ region, whereas the water molecule was initially located inside the buffer region $\mathbb{B}$. As the intermolecular distance increases beyond the $\mathbb{B}$ cutoff (5.0 Å), the water molecule leaves the buffer region and the $\mathbb{I}$-$\mathbb{O}$ coupling contribution becomes active. The transition occurs already at a $r_{\mathrm{MeOH}-O_{\mathrm{W}}}$ distance of 4.7 Å. This arises because the $\mathbb{B}$ assignment is based on the center of geometry of the water molecule, which crosses the boundary before the oxygen atom itself reaches the $\mathbb{B}$ cutoff.

As shown in Figure S1, the isolated BuRQM contribution, $V^{\mathrm{BuRQM}}_{\mathbb{I}+\Delta\mathbb{B}}$, exhibits the expected discontinuity at the buffer boundary due to the abrupt change in the QM region assignment. However, this discontinuity is compensated by the corresponding MM interaction term, $V^{\mathrm{MM}}_{\mathbb{O}(\mathbb{I}+\mathbb{B})}$, such that the total interaction energy remains continuous and smooth across the transition region.

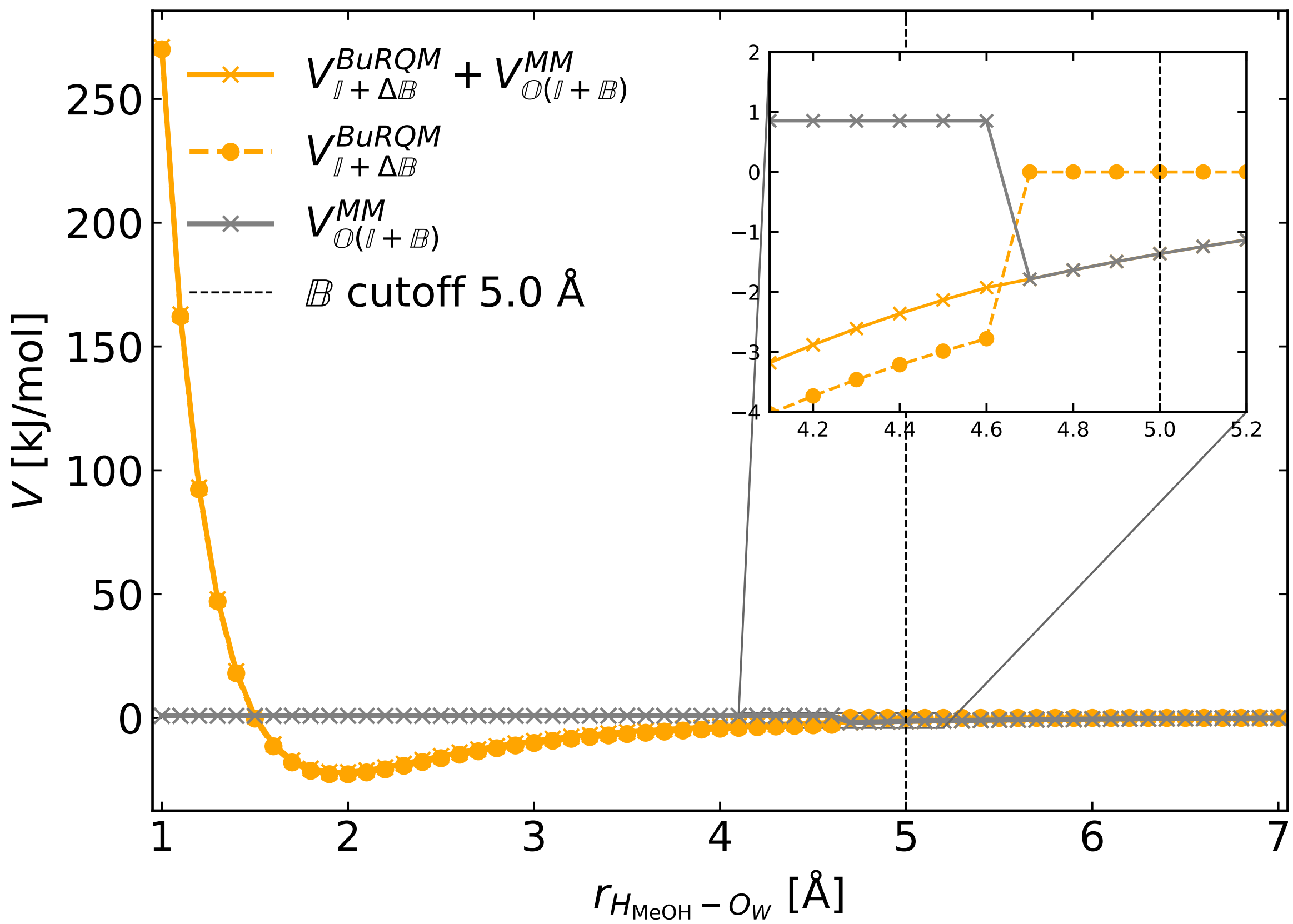

Figure S1: Potential energy surface for the dissociation of an isolated methanol–water hydrogen bond in vacuum. The methanol molecule belongs to the $\mathbb{I}$ region, while the water molecule transitions from the $\mathbb{B}$ into the $\mathbb{O}$ as the intermolecular distance increases. The vertical dashed line indicates the $\mathbb{B}$ cutoff of 5.0 Å. The inset highlights the transition region around the cutoff. All energy contributions were shifted such that their values are zero at the largest intermolecular separation (7.0 Å), facilitating a direct comparison of the dissociation profiles.

Methanol-Water Mixtures: Additional Information

Structure factor: Calculation details

To analyze the structural behaviour of water-methanol mixtures in BuRNN the total structure factor $S(q)$ was computed from the partial structure functions S_{ij}. The partial structure functions are the Fourier transforms of the intra- and intermolecular site-site radial distribution functions (RDFs) given by Equation S1:

$$S_{ij}(q) = 1 + 4\pi\rho_N \int_0^\infty r^2 \left(g_{ij}(r) - 1\right) \frac{\sin(qr)}{qr}\, dr, \tag{S1}$$

where i-indices correspond to $\mathbb{I}$ atoms (either methanol or water atoms) and j runs over all atoms. The site-site RDFs were constructed using 126 grid points evenly spaced up to a maximum distance of 12 Å. ρ_N denotes the total number density of atoms (given in Å^{-3}). The total scattering structure factor for a given experiment is the weighted sum of the partial site-site structure functions given by Equation S2:

$$S(q) = \sum_{i=1}^{n} \sum_{j=i}^{n} w_{ij}(q)\, S_{ij}(q), \tag{S2}$$

where n is the number of atom types defined of the system and in this study compromises a 6-site methanol model and a 3-site water model. The weights are defined by Equation S3:

$$w_{ij}(q) = \frac{c_i c_j f_i(q) f_j(q)}{\left(\sum_k c_k f_k(q)\right)^2} \left(2 - \delta_{ij}\right). \tag{S3}$$

These coefficients depend on the concentration of atoms of type i as $c_i = N_i / \sum_i N_i$, the sum runs over all types of atoms. δ_{ij} is the Kronecker symbol, evaluating to 1 if $i = j$ and 0 otherwise. The form-factors, $f_i(q)$ are taken in the form of a sum of five Gaussians with the coefficients (a, b, c) tabulated for different atoms as given by Equation S4:[S1]

$$f_i(q) = \sum_{\alpha=1}^{5} a_\alpha(i)\, \exp\left(-b_\alpha(i)\, q^2\right) + c_i. \tag{S4}$$

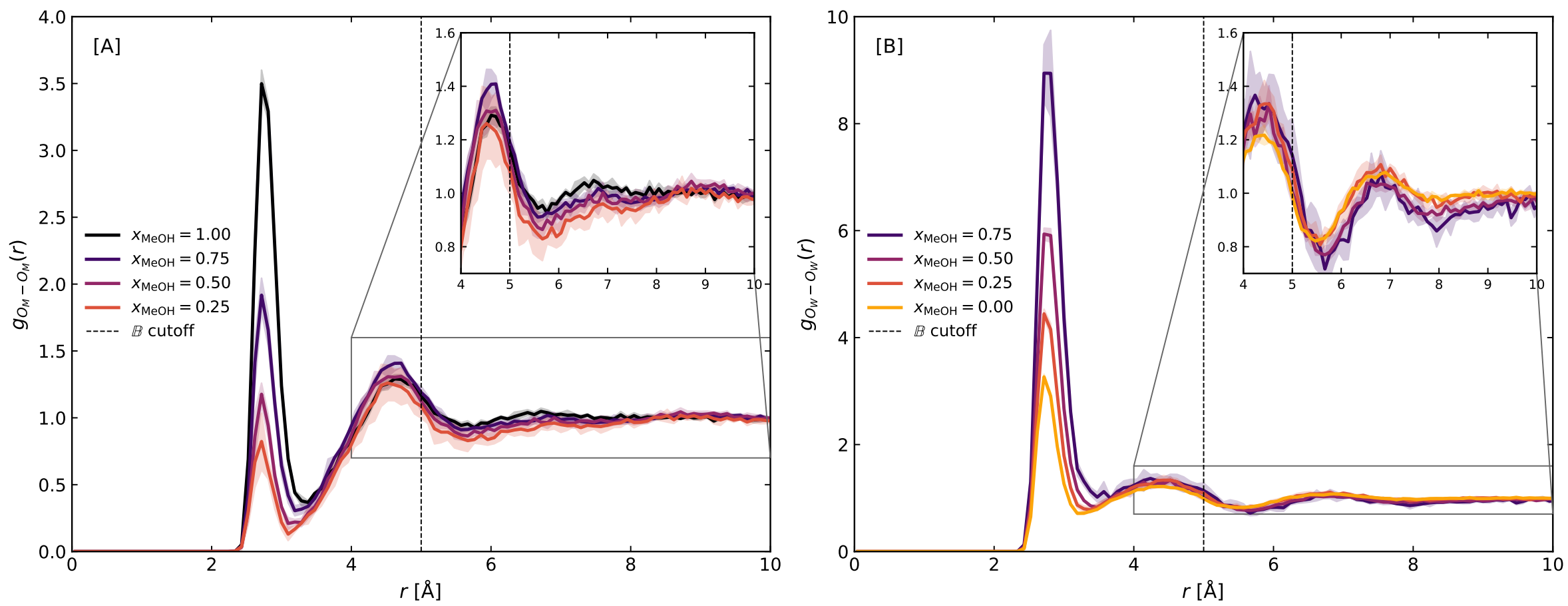

Figure S2: Oxygen-Oxygen RDF obtained from the BuRNN complete model simulations. Panel [A] shows the data for the methanol oxygen - methanol oxygen RDF data $g_{O_\mathrm{M}-O_\mathrm{M}}(r)$, and panel [B] shows the water oxygen - water oxygen RDF, $g_{O_\mathrm{W}-O_\mathrm{W}}(r)$. Shaded regions indicate the standard deviation over the analyzed trajectory blocks, used in the block averaging of the structure factors. The vertical dashed line indicates the $\mathbb{B}$ cutoff at 5.0 Å. The insets highlight the region around and beyond the cutoff.

Power spectrum

The vibrational power spectra were calculated for the methanol mole fractions in Table 1, with $\mathbb{I}$ consisting of a methanol molecule. For each composition three independent NVT production simulations of 100 ps were performed using the same thermostat and simulation settings as described in methods section for the Methanol-Water Mixtures. The vibrational spectra were obtained from Fourier transforms of the corresponding time-correlation functions calculated from the C-O bond distance, O-H bond distance, three C-H bond distances and the C-O-H bond angle. For each property and composition the spectra were averaged over the three replicas and normalized by the integrated spectral intensity. For visualization purposes, each spectrum was smoothed using a one-dimensional Gaussian filter with $\sigma = 0.5$ data point prior to replica averaging.

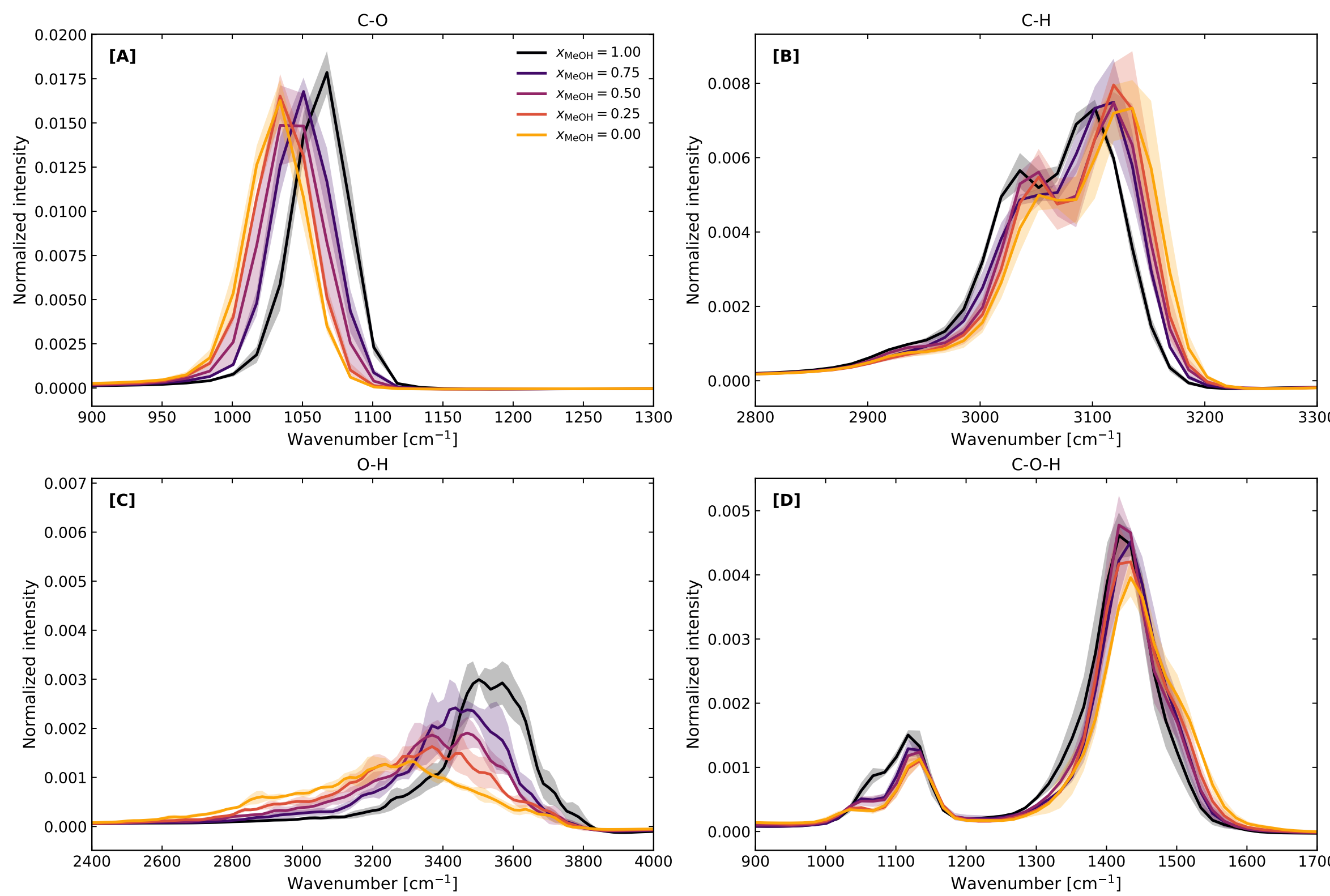

Figure S3: Power spectra obtained from BuRNN simulations for methanol internal vibrational modes at different methanol mole fractions x_{MeOH}. Panels show spectra for [A] C–O stretching, [B] C–H stretching, [C] O–H stretching, and [D] C–O–H angle vibrations. Shaded regions correspond to the standard deviation obtained from three independent replicas.

Computational Performance

To assess the computational efficiency of the BuRNN methodology, we benchmarked MM, BuRNN, QM/MM, and BuRQM simulations for the pure methanol system ($x_{\mathrm{MeOH}} = 1.00$), simulated with the same settings as described in the methods section of the methanol-water mixtures with a $\mathbb{B}$ cutoff of 5.0 Å. The QM/MM system only contained a single methanol molecule in the $\mathbb{I}$. Consequently, the reported QM/MM performance represents an optimisic estimation of the computational cost. The measured simulation performance is summarized in Table S1. BuRNN production simulations achieved a performance of 3670 ps/day, compared to 12.88 ps/day for QM/MM and only 0.12 ps/day for direct BuRQM simulations. Consequently, BuRNN accelerates the simulations by approximately two orders of magnitude relative to QM/MM and by more than four orders of magnitude relative to direct BuRQM simulations.

The final methanol BuRNN model was generated using an active-learning(AL) procedure consisting of a cumulative total of 16 148 000 MD steps, corresponding to approximately 8.1 ns of total sampling. During the AL procedure, 4749 QM reference configurations were evaluated. On average, each QM calculation for the $\mathbb{I} + \mathbb{B}$ region required approximately 30 minutes on 4 CPU cores. Including all AL MD simulations, QM reference calculations, and MLIP training iterations, the total wall-clock time required to generate the final BuRNN model was approximately 115 hours when exploiting parallel execution of up to 5 MD trajectories, 100 QM calculations, and 5 training runs.

In contrast, generating the same amount of sampling directly at the BuRQM level would require approximately 67 000 simulation days ($\sim$ 180 years) on the employed hardware based on the measured BuRQM performance of 0.12 ps/day. Accordingly, although the generation of the BuRNN model requires a substantial upfront computational investment, this cost is rapidly amortized once nanosecond-timescale production simulations are required.

Table S1: Performance measured with 8 cores on a NVIDIA GeForce RTX 5070 Ti with AMD Ryzen 9 9950X 16-Core Processor. The simulated system was the pure methanol composition $x_{MeOH} = 1.00$ simulated with the same settings as described in the methods section of the methanol-water mixtures. MM and BuRNN simulations were benchmarked over 100 ps, QM/MM simulations over 5 ps, and BuRQM simulations over 0.01 ps.

System	MM [ps/day]	BuRNN [ps/day]	QM/MM [ps/day]	BuRQM [ps/day]
MeOH	6000.00	3670.00	12.88	0.12

Solvated heme b

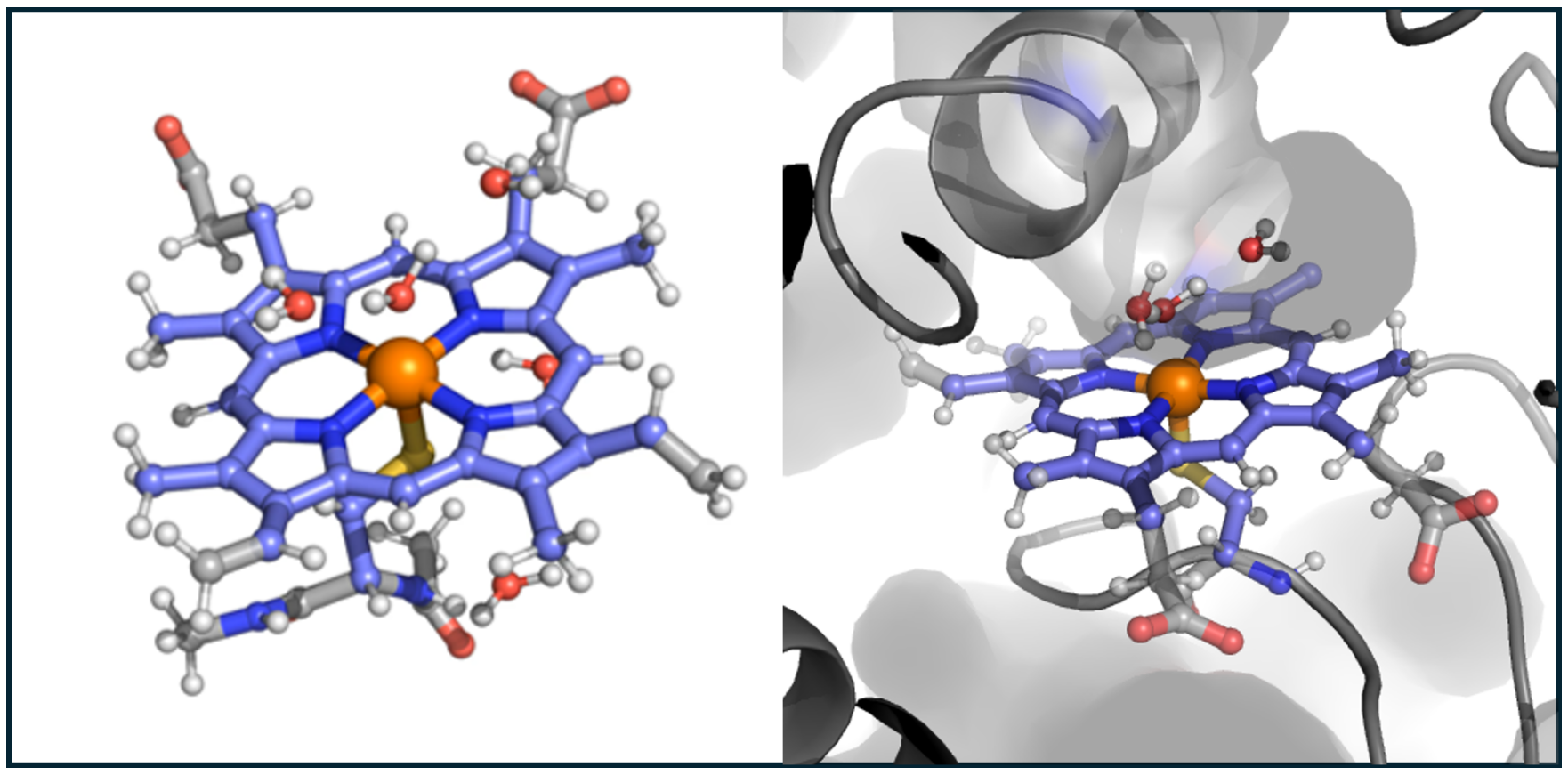

Figure S4: Representative structures of the heme b model used for BuRNN simulations. The $\mathbb{I}$ in (orange) comprises Fe(III), whereas the $\mathbb{B}$ statically included the porphyrin macrocycle and axial thiolate, and dynamically all water molecules within 5 Å of $\mathbb{I}$ (blue carbon atoms). The sidechains of the heme b are shown semi-transparent with grey carbon atoms to indicate the $\mathbb{O}$. The left panel illustrates the solvated heme b model while the right panel shows the same heme b model embedded in the CYP1A2 protein environment

Table S2: The equilibrium values of ξ_{Fe-O_W} and their corresponding force constants k as used for the active learning.

ξ_{Fe-O_W}	k [kJ/mol]
1.0	2500
0.9	20000
0.8	10000
0.6	2500
0.4	2500
0.2	2500
0.1	5000
0.0	15000

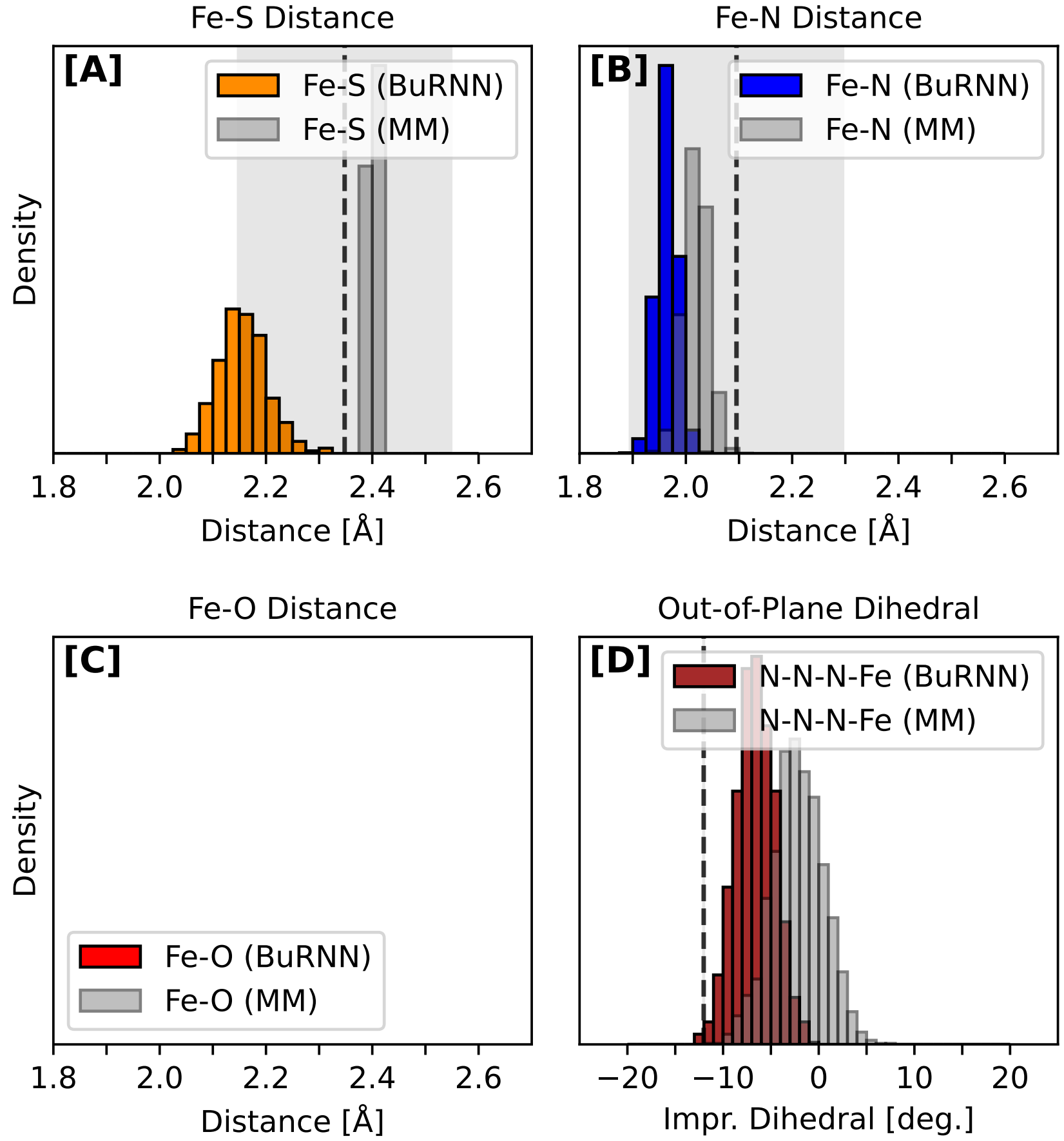

Figure S5: Comparison of geometric HEME parameters obtained from BuRNN and reference MM simulations at $\xi_{Fe-O_W} = 0$ in aqueous solution. Histograms show the distribution of [A] Fe-S bond distance, [B] mean of Fe-N(porphyrin) distances, [C] Fe-O distance of the closest water molecule, and [D] out-of-plane porphyrine distortion quantified by mean of the N-N-N-Fe improper dihedrals. BuRNN results are shown in color, while MM reference data are shown in grey. The vertical dashed lines indicate experimental values derived from the crystal structure (PDB ID: 2HI4). Grey shaded regions in panels A–C denote a ± 0.2 Å comparison window around the experimental bond distances to guide visual assessment of agreement.

WHAM Analysis and Uncertainty Estimation

The potential of mean force (PMF) along ξ_{Fe-O_W} was reconstructed using the weighted histogram analysis method.[S2] For W umbrella windows and histogram bin centers ξ_j, the unbiased probability p_j and window free-energy offsets F_i satisfy the Equations S5 and S6:

$$p_j = \frac{\sum_{i=1}^{W} n_{ij}}{\sum_{i=1}^{W} N_i \exp\left(\frac{F_i - U_i(\xi_j)}{k_B T}\right)}, \tag{S5}$$

$$F_i = -k_B T \ln\left(\sum_{j=1}^{N_{\text{bins}}} p_j \exp\left(-\frac{U_i(\xi_j)}{k_B T}\right)\right), \tag{S6}$$

where $U_i(\xi)$ is the bias potential in window i, n_{ij} counts observations in bin j from window i, and N_i is the total number of samples in window i. The PMF was computed as $\Delta G(\xi_j) = -k_B T \ln p_j + C$. Uncertainties were estimated using a moving-block bootstrap within each umbrella window. The coordination number was sampled every 0.5 ps. A block length of $10\tau_{\text{int}}$ was used, corresponding to 20 frames (10 ps) for the present simulations, where τ was estimated from the integrated autocorrelation times of ξ_{Fe-O_W}. For the final PMF uncertainty, 300 bootstrap resamples were generated and the standard deviation across resampled PMFs was reported. Histograms of ξ_{Fe-O_W} were computed using 168 bins over the interval $[0.000, 1.025]$.

Table S3: The equilibrium values of ξ_{Fe-O_W} and their corresponding force constants k as used for the Production runs.

ξ_{Fe-O_W}	k [kJ/mol]
1.00	250
0.95	30000
0.90	15000
0.85	7500
0.80	7500
0.75	7500
0.70	7500
0.65	5000
0.60	2500
0.55	2500
0.50	2500
0.45	2500
0.40	2500
0.35	2500
0.30	2500
0.25	2500
0.20	2500
0.15	2500
0.10	2500
0.05	2500
0.00	5000

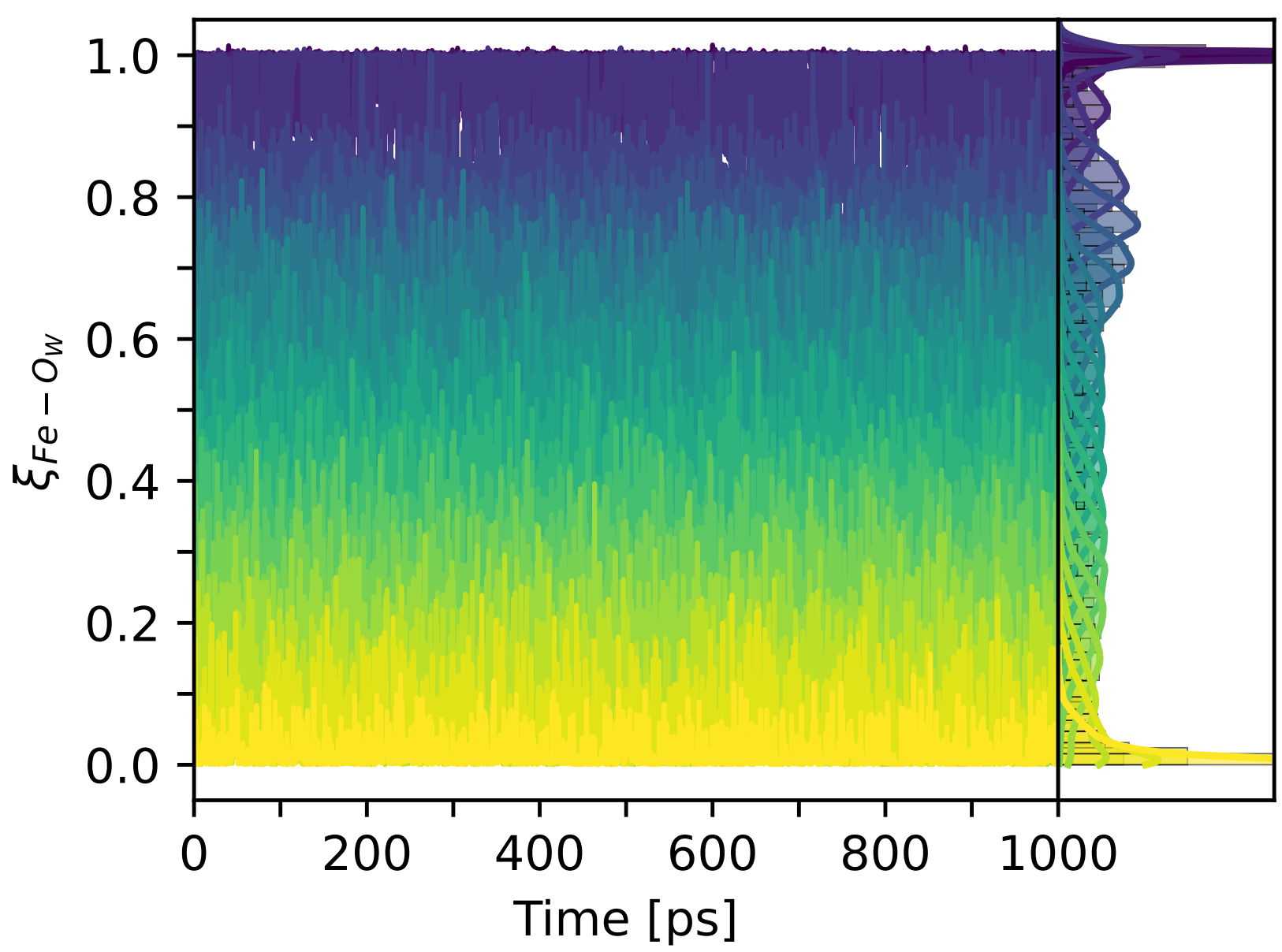

Figure S6: Time series of the coordination number ξ_{Fe-O_W} and its corresponding histogram distributions for the BuRNN umbrella sampling simulations

CYP1A2: Additional Information

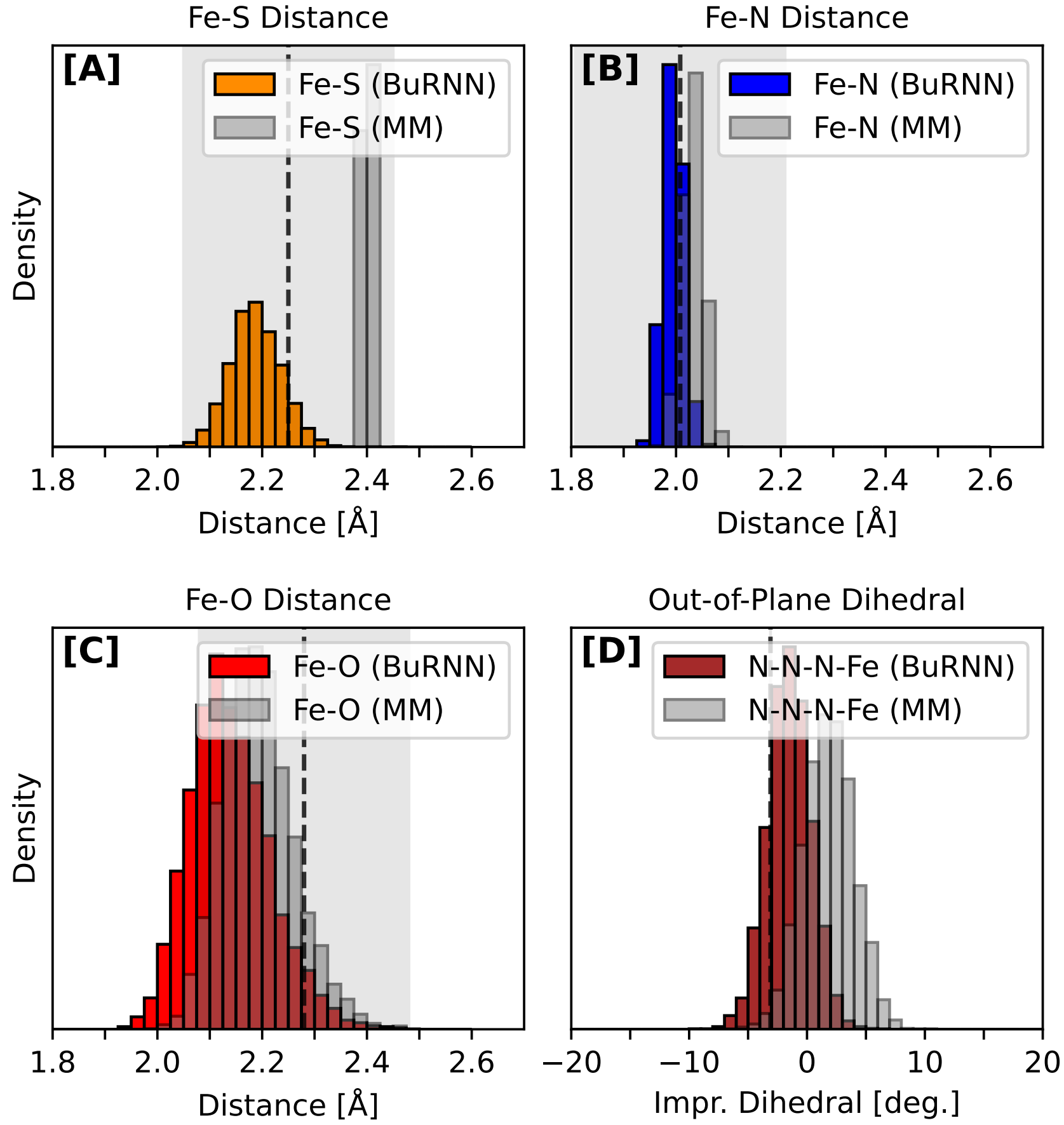

Figure S7: Comparison of geometric HEME parameters obtained from BuRNN and reference MM simulations in the CYP1A2 system. Histograms show the distribution of [A] Fe-S bond distance, [B] mean of Fe-N(porphyrin) distances, [C] Fe-O distance of the closest water molecule, and [D] out-of-plane porphyrine distortion quantified by mean of the N-N-N-Fe improper dihedrals. BuRNN results are shown in color, while MM reference data are shown in grey. The vertical dashed lines indicate experimental values derived from the crystal structure (PDB ID: 1PHC). Grey shaded regions in panels A–C denote a ±0.2 Å comparison window around the experimental bond distances to guide visual assessment of agreement.

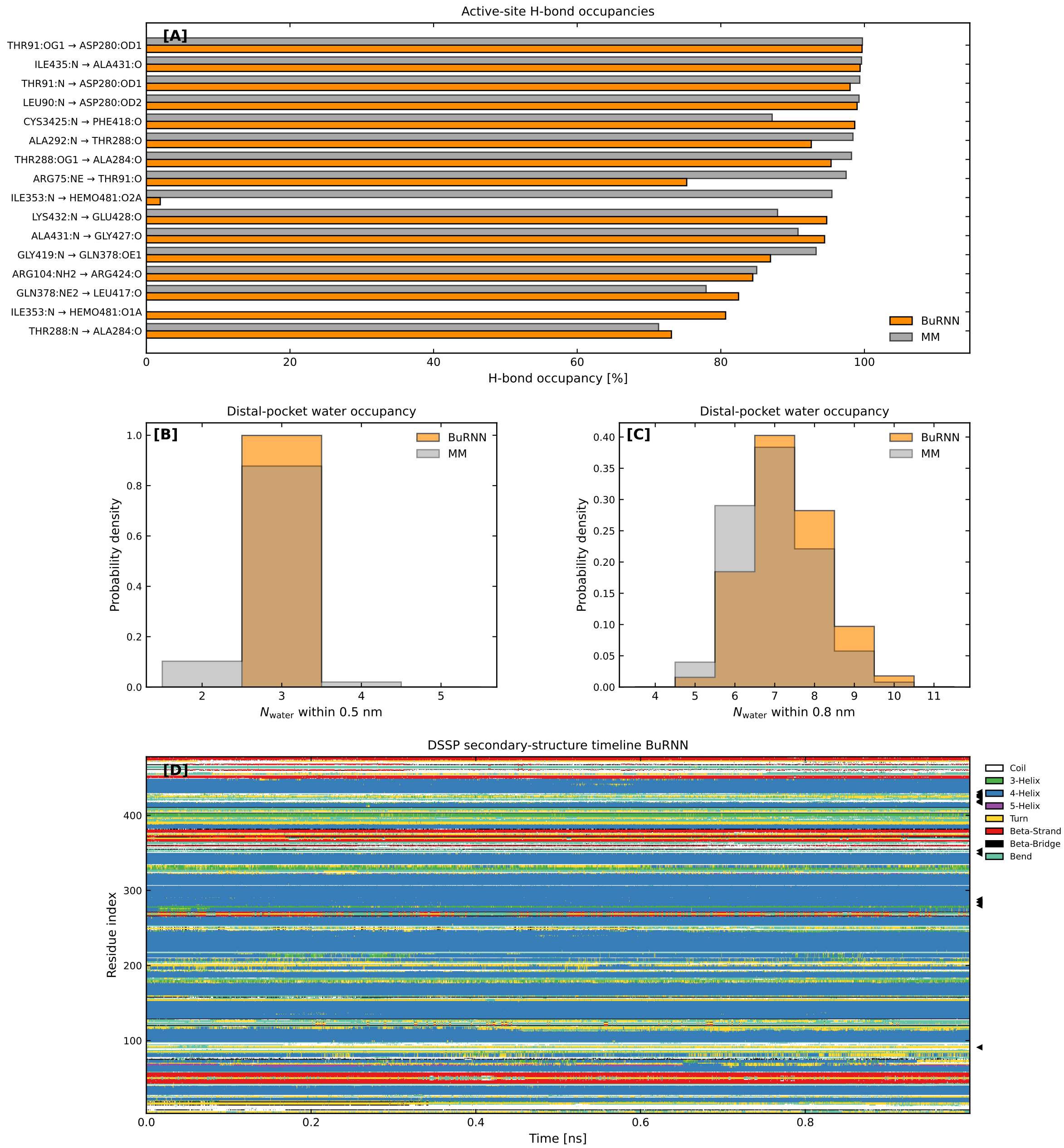

Figure S8: Characterization of the CYP1A2 protein stability and active-site environment. [A] Active-site hydrogen-bond occupancies for MM and BuRNN simulations. Note that the hydrogen bond from ILE353 to the Heme proprionate A, switches from atom O2A to atom O1A. [B] Distal-pocket water occupancy distributions using a 0.5 nm cutoff around the heme iron. [C] Distal-pocket water occupancy distributions using a 0.8 nm cutoff around the heme iron. [D] DSSP secondary-structure timeline for the BuRNN simulation. Active-site residues are indicated on the right-hand side.

References

(S1) Waasmaier, D.; Kirfel, A. New analytical scattering-factor functions for free atoms and ions. *Acta Crystallographica Section A* **1995**, *51*, 416–431.

(S2) Kumar, S.; Rosenberg, J. M.; Bouzida, D.; Swendsen, R. H.; Kollman, P. A. THE weighted histogram analysis method for free-energy calculations on biomolecules. I. The method. *Journal of Computational Chemistry* **1992**, *13*, 1011–1021.
